# Small nutrient molecules in fruit fuel efficient digestion and mutualism with plants in frugivorous bats

**DOI:** 10.1038/s41598-019-55915-z

**Published:** 2019-12-18

**Authors:** S. Andrea Moreno, Mariana Gelambi, Alejandro Biganzoli, Jesús Molinari

**Affiliations:** Departamento de Biología, Facultad de Ciencias, Universidad de Los Andes, Mérida, 05101 Venezuela

**Keywords:** Ecophysiology, Coevolution, Animal physiology

## Abstract

Frugivorous bats often possess short intestines, and digest rapidly. These characters are thought to be weight-saving adaptations for flight. The hypothesis that they limit digestive efficiency was tested by assaying glucose and protein in fecal samples of a free-ranging bat, and in fruit of its main food plant. To assure the correct calculation of digestive efficiencies, seeds were used as a mass marker for nutrients in fruit and feces. Glucose represents 32.86%, and protein 0.65%, of the nutrient content of fruit. Digestive efficiencies for these nutrients respectively are 92.46% and 84.44%, clearly negating the hypothesis for glucose. Few studies have quantified protein in fruit. Instead, “crude protein”, a dietary parameter solely based on nitrogen determinations, is used as a surrogate of protein content. This study shows that, for fruit consumed by bats, crude protein estimates typically are much greater than true protein values, implying that a large fraction of the crude protein reported in previous studies consists of free amino acids. The rapid digestion of frugivores has the potential to limit protein digestion, thus it may require free amino acids for efficient assimilation of nitrogen; therefore, the crude protein approach is inadequate for the fruit that they consume because it does not differentiate free amino acids from protein. Adding simple sugars and free amino acids, instead of protein, to fruit reduce metabolic costs for plants. Direct assimilation of these small nutrient molecules increases digestive and foraging efficiencies. Both factors contribute to the persistence of the mutualism between plants and frugivores, with community-wide repercussions.

## Introduction

One crucial aspect of animal foraging is the quantity of energy and nutrients obtained for metabolic use per unit time^1^. If expressed relatively to ingested food, this quantity is known as digestive efficiency, defined as the percentage of energy or nutrients that is assimilated after digestion^2^. Digestive efficiency influences the flexibility of the diet, hence the niche breadth, resource partitioning, and coexistence of species^3,4^.

The evolution of flight in bats and birds has been accompanied by modifications in their digestive systems, manifest in rapid digestion and short intestines, thought to have been selected to reduce body mass^5^. In the case of frugivorous birds, the relationship between digestion time, intestine length, and digestive efficiency has received considerable attention^5–8^. In the case of frugivorous bats, less information exists^5^.

In the tropical forests of South and Central America, bats of the genus *Carollia* (Phyllostomidae) are numerically prominent members of mammal communities^9^. Fruit of plants of the genus *Piper* (Piperaceae) are the staple food for these bats^10,11^. In this study, we determine the digestive efficiency of free-ranging silky short-tailed bats, *Carollia brevicauda*, that had fed on fruit of the spiked pepper, *Piper aduncum*. We do so by comparing the concentration of glucose and protein in ripe fruit with that of its digested remains in the form of fecal samples.

To avoid the miscalculation of digestive efficiencies caused by conventional gram-to-gram comparisons, we use ingested seeds as a mass marker for nutrients in both pulp and feces. We test the hypothesis that the short intestines and rapid digestion of frugivorous bats limit digestive efficiency for soluble sugars and protein, as observed in some birds^4^. In doing so, we use our data for *C. brevicauda* and *P. aduncum*, as well as data extracted from literature on the intestine lengths, gastrointestinal transit times, and digestive efficiencies of bats, and on the nutritional properties of fruit. The findings lead to question the use of nitrogen assays to infer the so-called “crude protein” content of fruit, as a surrogate of true protein content, in studies involving frugivorous animals.

## Results

### Nutrient composition and assimilation

**Figure 1:**
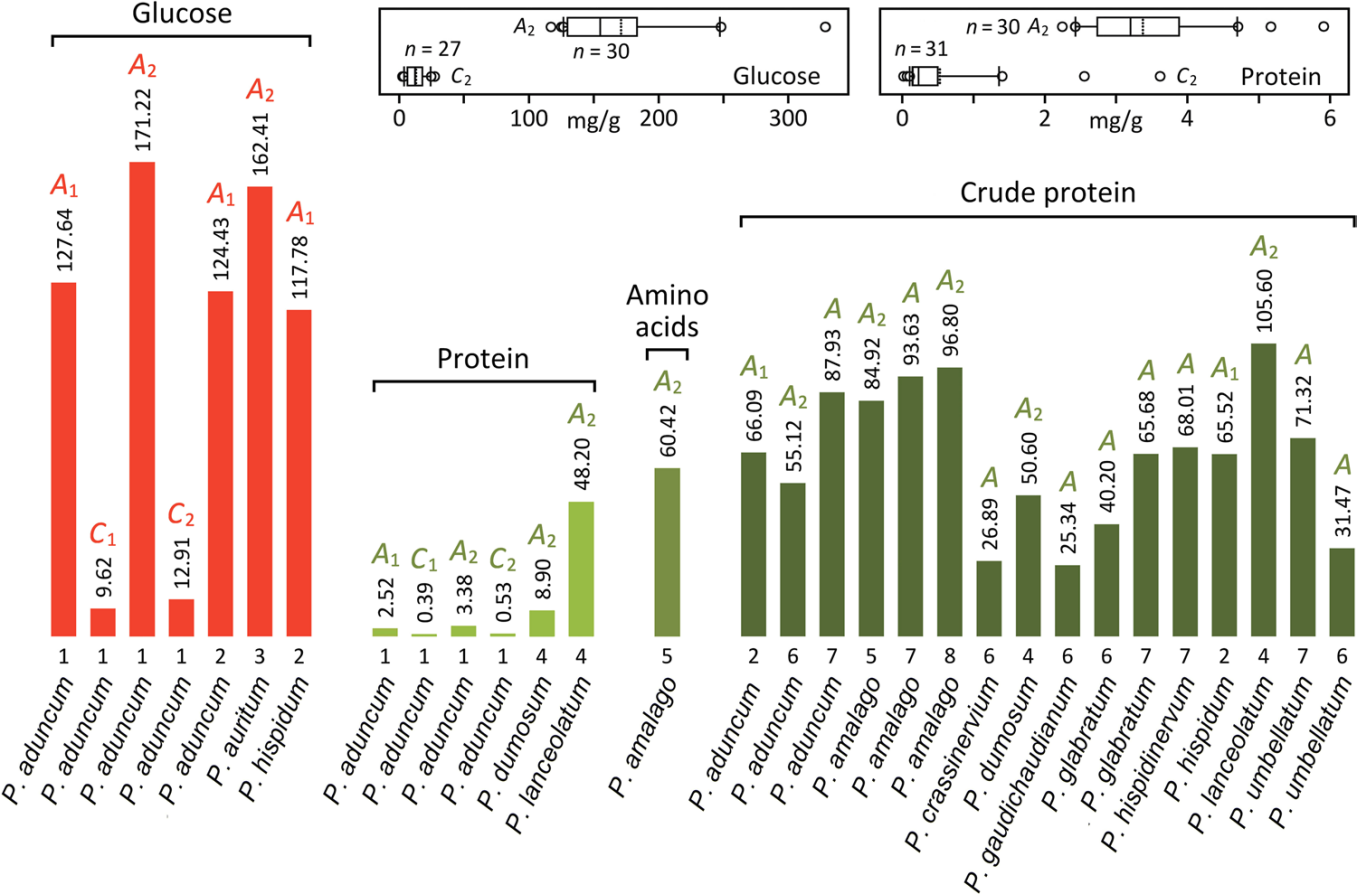
Glucose, protein, amino acid, and crude protein (N × 4.4) content of undigested and digested pulp of ripe fruit of *Piper* species used by bats of the genus *Carollia*: (**a**) vertical bar chart, comparison of 11 species of *Piper*. Bars represent the nutrient content (mg/g) of dry samples. Numbers above bars indicate their height. Numbers below bars indicate data sources. *A*_1_, glucose/protein in whole pulp; *C*_1_, glucose/protein in whole feces; *A*_2_, glucose/protein in pulp without seeds; *C*_2_, glucose/protein in feces without seeds; *A*, either *A*_1_ or *A*_2_ (not specified in data source); (**b**) box-and-whisker plots, data distribution (*A*_2_, *C*_2_) for *Piper aduncum* (this study). Vertical solid and dotted lines within boxes represent, respectively, medians and means. Boxes span 50% of data points. Horizontal lines span 80% of data points. Empty circles represent potential outliers; (**c**) highlights, quantifications were performed through enzymatic analyses (glucose), Bradford assays (protein), hydrolyzed pulp analyses (amino acids), and Kjeldahl or Dumas nitrogen assays (crude protein). Hydrolyzed pulp analyses do not differentiate free amino acids from peptide/protein amino acids. Nitrogen assays do not differentiate proteinaceous (forming free amino acids, or peptides/protein) from non-proteinaceous nitrogen. Amino acids (*P. amalago*) were made quantitatively comparable to protein and crude protein through multiplication of total amino acid nitrogen by a conversion factor (5.70)^18^; (**d**) data sources, 1, this study; 2, Kelm *et al*.^13^; 3, Becker *et al*.^73^; 4, Ripperger *et al*.^22^; 5, Herbst^18^; 6, Batista *et al*.^12^; 7, Ricardo^74^; 8, Dinerstein^75^.

Descriptive statistics (mg/g) for our data (Fig. 1), expressed as mean ± standard deviation (minimum–maximum) [sample size], are (for the equations defining *A*_1_, *A*_2_, *C*_1_, and *C*_2_, see Methods): glucose in whole pulp (*A*_1_), 127.64 ± 40.77 (87.27–244.95) [30]; glucose in whole feces (*C*_1_), 9.62 ± 5.31 (1.81–20.40) [27]; protein in whole pulp (*A*_1_), 2.52 ± 0.66 (1.67–4.41) [30]; protein in whole feces (*C*_1_), 0.39 ± 0.57 (0.02–2.70) [31]; glucose in pulp without seeds (*A*_2_), 171.22 ± 54.69 (117.06–328.59) [30]; glucose in feces without seeds (*C*_2_), 12.91 ± 7.13 (2.43–27.36) [27]; protein in pulp without seeds (*A*_2_), 3.38 ± 0.88 (2.24–5.91) [30]; protein in feces without seeds (*C*_2_), 0.53 ± 0.77 (0.02–3.62) [31].

We used Mann-Whitney *U* tests to compare samples without seeds (*A*_2_, *C*_2_): (a) glucose content, pulp vs. feces; (b) protein content, pulp vs. feces; (c) glucose/protein content, pulp vs. pulp, and feces vs. feces. To prevent Type I errors as a consequence of carrying out multiple tests, we used the Bonferroni correction: the desired significance level (*α* = 0.01) was divided by the number of tests (= 4). Thus, the Bonferroni critical value is 0.0025. In each of the comparisons (*U* = 0.00, 27.00, 0.00, 3.00), the *p*-value was much smaller than 0.0025.

The fruit of *Piper* species is much richer in glucose than in proteinaceous (protein, peptides, free amino acids) nutrients (Fig. 1). Our glucose values for pulp (*A*_1,_ *A*_2_) are similar to those previously reported for *P. aduncum* and *P. hispidum* (*A*_1,_ *A*_2_). Our glucose values for pulp (*A*_1,_ *A*_2_) are 13.3 times greater than those for feces (*C*_1,_ *C*_2_).

Limiting the attention to protein, our value for pulp (*A*_2_) is 2.6 times lower than that of *P. dumosum* (*A*_2_), and 14.3 times lower than that of *P. lanceolatum* (*A*_2_) (Fig. 1), hinting to substantial variability among species of *Piper* in nutritional content of fruit. Our protein value for pulp (*A*_1,_ *A*_2_) is 6.4–6.5 times greater than that for feces (*C*_1,_ *C*_2_).

Protein values (*A*_1_, *A*_2_) are generally lower than the single amino acids value (*A*_2_), and the crude protein values (*A*, *A*_1_, *A*_2_) (Fig. 1). All the low crude protein values (*A*) come from a single study^12^. For *P. aduncum*, *P. dumosum*, and *P. lanceolatum*, protein values (*A*_2_) are lower than crude protein values. Within *P. amalago*, the single amino acids value is lower than the crude protein values.

The protein content (*A*_2_) of the fruit of *P. aduncum*, *P. dumosum*, and *P. lanceolatum* (mean = 20.16 mg/g; range = 3.38–48.20 mg/g) is lower (Fig. 1) than: (a) the crude protein content of the fruit of 7 species of *Piper* (mean = 66.0 mg/g; range = 34.32–92.84 mg/g) eaten by the frugivorous bats, *Dermanura tolteca* and *Sturnira burtonlimi*; (b) the crude protein content of the fruit of 103 species of other plant genera (mean = 50.48 mg/g; range = 4.22–147.80 mg/g) eaten by phyllostomid and pteropodid bats (Table S1, Supplementary information).

In *P. amalago*, proteinaceous nitrogen represents 54.92% of total nitrogen, whereas in of *P. aduncum*, *P. dumosum*, and *P. lanceolatum* protein-only nitrogen respectively represents around 3.28, 13.57, and 35.25% of total nitrogen (Table S2).

### Digestive efficiency

To determine digestive efficiencies (column D; Table 1), we used data on nutrient assimilation expressed as ratios (columns *P* and *F*; Table 1) of seedless items (dry matter, glucose, protein, other nutrients) to seed mass. We calculated three measures of digestive efficiency (Table 1). The first one, which might called dry matter, or whole pulp digestibility, refers to the fraction of the fruit pulp that is assimilated by bats. The other two refer to the fractions of the glucose and protein present in fruit that are assimilated by bats.

**Table 1:** Estimated nutrient absorption from ripe fruit pulp of the spiked pepper, *Piper aduncum*, by silky short-tailed bats, *Carollia brevicauda*.

	Pulp (*P*)	Feces (*F*)	Digested nutrients (*N* = *P* - *F*)	Digestive efficiency (*D* = 100 × *N*/ *P*)
Dry matter^a^	2928.76	1518.70	1410.06	48.15
Glucose	501.47	37.80	463.67	92.46
Protein	9.90	1.54	8.36	84.44
Other nutrients	1014.53^d^	76.50^e^	938.03^b^	92.46^c^

The equations used for calculations (change in dry matter of feces with respect to dry matter in intact pulp, in both cases excluding seeds) are indicated. Masses (dry weight) of pulp, feces, and digested nutrients are expressed in milligrams per gram of seeds. Digestive efficiencies are expressed as percentages. Use of seeds as a mass marker in both pulp and feces assures correct calculation of digestive efficiencies. Values in the “other nutrients” row were guessed as indicated in the footnote. Descriptive statistics for nutrient determinations are given in Results.

^a^Excludes seeds. Includes glucose, protein, other nutrients, fiber, and indigestible compounds.

^b^Calculated as *N* = total digested nutrients - digested glucose - digested protein = 1410.06 − 463.67 − 8.36.

^c^Assumes “other nutrients” to be mainly fructose, sucrose, and free amino-acids, with a joint digestive efficiency equal to that of glucose: *D* = 92.46.

^d^Estimated as *P* = 100 × *N*/*D* = 100 × 938.03/92.46.

^e^Estimated as *F* = *P* − *N* = 1014.53 − 938.03.

Glucose = 100 × 501.47/(501.47 + 9.90 + 1014.53) = 32.86% of nutrient content.

Protein = 100 × 9.90/(501.47 + 9.90 + 1014.53) = 0.65% of nutrient content.

We reverse-calculated the quantities of “other nutrients” in fruit and feces (Table 1). Our hierarchy of digestive efficiencies is glucose > protein > whole fruit. Figures in columns *P* and *F* (Table 1) rest on the assumption that “other nutrients” are mainly fructose, sucrose, and free amino acids, which for the reason of being small molecules, should be assimilated with a joint digestive efficiency as high as that of glucose, which is also a small molecule. Should the digestive efficiency of “other nutrients” be lower than that of glucose, the quantities of “other nutrients” in fruit and feces (columns *P* and *F*; Table 1) would be greater, and the proportion of glucose and protein in total nutrients would be smaller.

In terms of dry weight, glucose and protein have been reported to respectively represent 30.35% and 16.14% of the nutrient content of *P. aduncum* fruit, the other nutrients (53.51%) quantified in that study^13^ being fructose (36.34%), sucrose (3.41%), starch (13.52%), and lipids (0.24%). Except for the very low protein content, our results (Table 1) are similar.

## Discussion

The Kjeldahl and Dumas nitrogen determination methods, combined with a conversion factor to express nitrogen content as crude protein, are routinely used for the nutritional assessment of cultivated fruit^14–16^, thus they have also been adopted in most studies on wild fruit (Table S1). The pervasive overestimation of fruit protein inherent to the 6.25 conversion factor has led to the proposal of more conservative factors^17–21^, ranging from 3.12 to 5.64. No matter the conversion factor, extrapolation from nitrogen content is likely to produce erroneous estimates of protein content because the proportion of non-amino acid nitrogen varies greatly according to fruit species, causing crude protein values to change without relation to true protein^17,21^. A case in point (Fig. 1) based on data from a single study^22^: the crude protein to protein ratio in fruit is much greater in *Piper dumosum* (5.69 times) than in *P. lanceolatum* (2.19 times). Herbst^18^ partly circumvented these problems by quantifying amino acids from the hydrolyzed fruit pulp of *P. amalago* (Fig. 1). Though this method allows a reliable quantification of proteinaceous content, the ratio of protein to free amino acids, which may also vary according to plant species, stays unknown.

For fruit of *Piper* species, despite the use of a conservative (N × 4.4)^17^ conversion factor, crude protein values tend to be much greater than protein values (Fig. 1). Such crude values are in the same range as those reported for fruit of many other bat-dispersed plant genera (Table S1). The *Piper* data imply true protein to be much less abundant in fruit than other nitrogen compounds. Broader comparisons are not possible because direct protein determination is seldom used to evaluate the nutritional composition of fruit. As in the case of *Piper*, some tropical fruit species have been reported (Table S3) to be high in crude protein content (mean = 74.49 mg/g; range = 10.75–141.30 mg/g), but low in true protein content (mean = 0.35 mg/g; range = 0.10–0.80 mg/g).

Our results should not be interpreted as indicating that the fruit of *P. aduncum* is low in proteinaceous content. Certainly, this fruit has little protein, but ample room remains for the presence of free amino acids. The question is, what is the proportion of these small molecules with respect to protein? The study of Herbst^18^ sheds light on the matter. His “protein” value (inferred from total amino acid content) for *P. amalago* is 17.88 times greater than ours for *P. aduncum* (Fig. 1). However, as already pointed out, his quantification method does not differentiate free amino acids from those obtained through the digestion of protein. Based on our results, we assume that most of his “protein” consisted of free amino acids, and possibly also small peptides.

A strong predominance of free amino acids over protein may be an overlooked and functionally highly consequential property of fruit. Digestion is a time-dependent process: generally, the longer the food is retained in the gastrointestinal tract, the greater the nutrient assimilation^23,24^. The very rapid digestion of frugivores (Table S4) may limit protein hydrolysis, thus it may require free amino acids for efficient assimilation of nitrogen. It follows that the crude protein approach is inadequate for frugivores because it does not differentiate free amino acids from protein. On the contrary, the slow digestion of omnivores and herbivores (Table S4) may favor protein hydrolysis, thus assimilation of peptide amino acids. The success of the crude protein approach can be explained because the question of whether nitrogen is in the form of free amino acids or protein amino acids has little nutritional relevance for animals with a slow digestion, therefore capable of greater protein hydrolysis, such as humans.

Fruit is a sink for free amino acids transported by the phloem^25^. Free amino acids can be sweet, bitter, or sour^26^, and are the precursors for volatile compounds that enhance the flavor and aroma of ripe fruit^27,28^. Therefore, free amino acids are expected to be present in the pulp of most fleshy fruit not only for their nutritional value, but also because they: (1) are delivered there; (2) convey gustatory information on the quality of pulp; (3) produce olfactory cues that attract frugivores, thus ensuring seed dispersal.

The early prediction, based on the rapid digestion of frugivorous birds, that free amino acids, along with simple sugars, should be the major nutrients of the pulp of fleshy fruits^29^ has been corroborated multiple times. A study^21^ of the fruit of 18 species of bird-dispersed plants from North America has shown that the mass of free amino acids (mean = 8.20 mg/g) in pulp exceeds that of protein (mean = 6.40 mg/g) in 12 cases, and that of non-proteinaceous nitrogen (mean = 2.70 mg/g) in all cases. In fruit of the genus *Citrus*, free amino acids typically represent 70% of the mass of nitrogen compounds^30^; whereas in fruit of the genus *Annona*, the mass of free amino acids is 5.57–25.66 mg/g, depending on the species^31^. Fruit of both genera are eaten by pteropodid bats throughout their range^32^. In cultivated fruit in general, free amino acids average ~50% of the mass of soluble nitrogen compounds^33^. Several species of tropical fruit^34–36^, including one species of *Piper*^37^, have been shown to be diverse and rich in free amino acids. In the fruit pulp of 14 bat-dispersed species of *Ficus* from Panama, the joint mass of 19 free amino acids averages 40.82 mg/g, and that of crude protein (N × 4.4) averages 39.42 mg/g^38^, implying that free amino acids are the vastly predominant, if not the only, nitrogen compounds in such pulp. From a mutualistic perspective, the latter example makes sense because bats do not ingest the pulp of *Ficus* fruit, which is spongy and highly fibrous, but instead swallow the juice obtained by biting with great force small chunks, which they later spit as dry pellets^39^.

The digestive efficiency of small mammals can vary greatly depending on diet. Fibrous (cellulose-rich) and hard (e.g., chitin-rich) foods are respectively more difficult to digest than non-fibrous and soft foods: accordingly, in the primarily herbivorous microtine rodents, the average digestive efficiencies for energy are 54% (monocotyledon shoots), 74% (dicotyledon leaves and stems), and 89% (seeds and garden vegetables)^40^; whereas in the primarily insectivorous shrews, the digestive efficiencies for carbon are 46.8–61.7% (adult beetles), 70.7–83.3% (ant pupae), 76.5–85.3% (sawfly cocoons), and 86.0–93.0% (fish)^41^. We consider whether this type of variation is observed in bats (note that the mealworms often used as experimental bat food are about as hard as adult beetles^42^). Previous studies (Table S5) on the digestive efficiencies of bats (6 frugivorous, 1 hematophagous, 11 insectivorous, and 2 nectarivorous species) have mainly dealt with energy and dry matter. Few have considered sugars or true protein.

The digestive efficiency for glucose of *C. brevicauda* eating *P. aduncum* is high (92.46%; Table 1) and within the range (89.2–98.7%; Table S5) of digestive efficiencies for sugars reported for frugivorous bats. Assimilation of soluble sugars from fruit naturally included in their diets by frugivorous birds typically is 86.0–99.0%^43–46^. Therefore, similarly to their avian counterparts, frugivorous bats appear to be nearly as efficient at assimilating soluble sugars as nectarivorous species.

The digestive efficiencies for energy (mean = 83.4%; range = 48.0–99.0%; Table S5) and protein (84.44%; Table 1) are lower than those for glucose (Table 1) and sugars in general (mean = 93.0%; range = 86.6–99.0%; Table S5). A reduction of the digestive efficiency for energy is expected if the computations include compounds that may fail to be fully degraded during digestion, such as poly and oligosaccharides, protein, and lipids. A reduction of the digestive efficiency for protein would be expected because, in contrast to simple sugars, it needs to be hydrolyzed before assimilation. However, the strictly hematophagous vampire bat, *Desmodus rotundus*, achieves a high digestive efficiency for protein (92.9%; Table S5), likely as a result of a digestive specialization to process a homogeneous, protein-rich, and fiber-poor food.

The digestive efficiencies for nitrogen and protein are often deemed comparable. This is incorrect because a large fraction of nitrogen in food can be non-proteinaceous, thus of little nutritional value^17,47^. Apparent digestive efficiencies for nitrogen decrease as non-proteinaceous nitrogen increases. In carnivorous mammals fed on horse meat, the efficiency is nearly as high (mean = 89.6%, range = 87.5–92.4%)^48^ as that of vampire bats (Table S5). In carnivorous birds fed on whole prey (shrimp, squid, fish, chicks), it is lower (mean = 76.3%; range = 68.3–82.0%)^49^. In frugivorous bats fed on fruit or fruit mixtures it is yet lower (mean = 58.6%; range = 49.0–68.4; Table S5). In frugivorous birds, very low efficiencies have been reported (26.0–41.0%)^50^ probably because of a failure to exclude uric acid from the computations^47^. Despite their enormous popularity, nitrogen assays can shed little light on digestive efficiency for protein unless the non-proteinaceous content of food is known.

Because it is a complex mixture of nutrients and non-digestible components, dry matter can provide only indirect information on assimilatory capacity. We found the digestive efficiency for dry matter to be lower (48.15%; Table 1) than reported for other bat species on various diets (mean = 76.3%; range = 54.2–94.2%; Table S5). This should simply reflect that the fruit pulp of *P. aduncum* contains a high proportion of non-digestible components, including structural carbohydrates and non-proteinaceous nitrogen. The fruit of *Piper* species contain amides that accelerate the passage time of seeds^51^, and bat-attracting essential oils^52^. These compounds are not likely to be digested (feces retain the aroma of *P. aduncum* fruit), hence they may also decrease digestive efficiency for dry matter.

We thus have that the digestive efficiencies reported for bats (Table S5) are generally higher than those reported for small non-flying mammals, both herbivorous^40^ and insectivorous^41^. Barring nitrogen and dry matter, the only low value (48.0%; Table S5) for a bat corresponds to the primarily nectarivorous bat, *Glossophaga commissarisi*, feeding on fruit: the slender jaws, diminutive teeth, and very short (10.5–11.1 cm) intestines of this bat are inadequate to masticate and digest fruit efficiently^13,53^.

Are frugivorous bats capable of high digestive efficiencies while possessing short intestines, and retaining ingested food for a short time? Let SLL be the small and large intestine length, and TT be the gastrointestinal transit time (defined as the time for first appearance of ingested matter in feces^54^). Previous studies (Table S4) on bats (6 frugivorous, and 11 insectivorous species) and small non-flying mammals (15 insectivorous, and 8 herbivorous species) have shown that: (a) in frugivorous phyllostomid bats (body mass = 16.5–67.7 g), SLL and TT are both short (20.9–47.8 cm; 5.2–27.0 min); (b) in frugivorous pteropodid bats (599.3–703.8 g), despite SLL being much longer (163.0–169.0 cm), TT can also be short (25.3–27.0 min); (c) in insectivorous bats (5.4–27.2 g), despite SLL being short (9.9–25.5 cm), TT is much longer (55.0–204.0 min); (d) in insectivorous non-flying mammals, specifically shrew-sized dasyurids and shrews (2.5–55.0 g), both SLL and TT tend to be longer (11.0–46.1 cm; 25.0–96.0 min); and (e) in herbivorous rodents (44.8–566.5 g), SLL and TT are both much longer (59.8–357.8 cm; 45.0–738.0 min). Therefore, frugivorous bats do not necessarily possess a short SLL. The achievement of high digestive efficiencies despite their short TT is what makes them peculiar, but not unique because nectarivorous bats (SLL short, TT presumably short) feeding on nectar are known to possess an even greater (>99%)^55^ digestive efficiency (Table S5).

In conclusion, contrary to our initial hypothesis, short intestines and rapid digestion do not limit the digestive efficiency of frugivorous bats. This may have as much to do with the nutritional composition of fruit, and with the microanatomy of the intestine^56^, as with the gross anatomy of the gastrointestinal tract. Our results support the previously known fact that fruit of *Piper* species is rich in soluble sugars. In addition, we have provided evidence suggesting that the “protein” found in this fruit should to a large extent be free amino acids, and perhaps also small peptides. The absorption of small nutrient molecules, such as simple sugars, free amino acids, and peptides, occurs directly through the paracellular and transcellular pathways^57,58^. The first is important in bats^56,59^. Food rich in small nutrient molecules should result in a fast and high assimilation, even with short intestines, as we observed.

The mutualism between plants and frugivores may have had a major impact on biological diversification during geological time, models the traits of participating organisms, and plays an important role in maintaining the structure of tropical forests^60–62^. A high concentration of free amino acids and soluble sugars in fruit is likely to be important for this mutualism. The probable effects, not necessarily in evolutionary order, are as follows: (1) by rewarding the frugivores with fruit containing simple nutrient molecules, plants avoid the greater metabolic costs of producing complex nutrient molecules^63^, thus becoming capable of allocating more resources to vegetative growth and to competition with other plants; (2) the foraging efficiency of frugivores increases by being rewarded with fruit containing simple nutrient molecules that can be readily assimilated through paracellular and transcellular absorption^29,56^; (3) the more efficient that frugivores are as foragers, the more of them that plants will be able to attract and sustain with a given biomass of fruit; (4) rapid assimilation of nutrients allows the frugivores to increase their feeding rates to acquire sufficient amounts of less abundant nutrients, such as free amino acids; (5) increased feeding rates augment fruit removal from plants, hence seed dispersal; (6) a high foraging efficiently is crucial for the survival of frugivores during climate-driven shortages of fruit^64,65^.

By enhancing the digestive efficiency and thus the foraging efficiency of frugivorous animals through the production of easily-assimilated nutrients, plants may produce more seeds relatively to reproductive effort, thus increasing the probability that some seeds arrive to adequate sites, while contributing to the persistence of the populations of their animal seed vectors despite fluctuations in the production of fruit. Therefore, on the part of plants, the production of fleshy fruit containing small nutrient molecules, coupled with, on the part of frugivores, direct assimilation of such nutrients, should be a crucial aspect of their mutualism, and have major community-wide repercussions.

## Methods

### Animal care

The animal and plant species sampled in this study are not regarded as endangered, threatened, or of special conservation concern by the International Union for Conservation of Nature (Red List of Threatened Species). The handling of bats were carried out in accordance with Venezuelan and international regulations (Ley para la Protección de la Fauna Doméstica Libre y en Cautiverio; Guidelines of the American Society of Mammalogists for the Use of Wild Mammals in Research) according to which field studies that do not harm wild animals are exempt from review by institutional animal care and use committees.

### Study organisms

The silky short-tailed bat, *Carollia brevicauda* (mean body mass = 17.9 g), is found in humid and mesic environments, mostly at medium elevations, throughout tropical South America^66^. In our study area, fruit of the spiked pepper, *Piper aduncum*, is the main component of its diet. The spiked pepper is a fast-growing pioneer plant, up to 8 m in height, thought to be the most invasive species of its genus; it is indigenous to tropical America, but has been introduced to Florida, many islands of the Pacific Ocean, and tropical Asia^67^. Its fruit is a cylindrical infructescence (mean dimensions = 142.6 × 4.6 mm; mean mass = 1.9 g; this study) formed by a fibrous axis surrounded by a fleshy pulp composed of hundreds of tiny drupelets, each containing a single seed (mean dimensions = 0.136 × 0.084 mm; this study; 7,750 seeds/g^68^).

### Collection of fruit and fecal samples

We carried out field work (2016–2018) in a cloud forest on the SE slope of the Sierra de La Culata, Venezuela. Our bat capture site (elevation, 2,200 m) is located at 8°40′22″N, 71°06′23″W. Our fruit collection area is within a radius of 300 m around this site. We captured bats by deploying mist nets across forest trails, and at the entrance of a small cave. We processed only adults. Immediately after capture, we placed each bat individually in a cloth bag with an internal plastic bottom, in which we held it for 60 min to make sure that any food remains present in its digestive tract would be defecated. After releasing the bat, we transferred each fecal sample to a vial, which we kept in an icebox until taken to the laboratory. We dried samples overnight at 50 °C. We discarded fecal samples containing insect fragments, or seeds other than those of *P. aduncum*.

### Biochemical analyses of samples

We quantified glucose and protein separately by splitting each sample into two fractions. We performed each quantification at least twice. Our sample sizes refer to the number of fruits or feces, not to the number of assays. To extract glucose, we followed Yelle^69^. To quantify glucose, we used a commercial assay kit (Wiener Laboratorios, Rosario, Argentina) based on the glucose oxidase method (2.5 mL assays). To extract protein, we followed Isaacson^70^. To quantify protein, we used a Bio-Rad protein assay kit (1 mL assays) based on the Bradford method, which detects peptides and proteins with molecular weights greater than 3–5 kDa (~27–45 average amino acid residues)^71^. For more details, see Supplementary information.

### Nutritional calculations

Frugivorous bats (*Artibeus*) feeding of fruit of plants of the genus *Ficus*, and nectarivorous bats (*Glossophaga*) feeding on fruit of *Piper auritum*, masticate slowly, and avoid swallowing part of the seeds. In contrast, species of *Carollia* masticate rapidly, and swallow all the seeds along with the pulp^39,72,73^. In preliminary trials, involving *C. brevicauda* placed in a flight cage, we verified that the whole fruit pulp of *P. aduncum* along with the seeds was ingested. We also found the density of seeds in feces to be uniform, indicating that their passage rate is the same as that of pulp. Therefore, for our study organisms, ingested seeds can be used as a mass marker for the quantification of selected nutrients in fruit and feces without the need to consider other nutrients and fiber.

Assimilation causes the concentration of nutrients to decrease, and the concentration of seeds to increase, in feces with respect to pulp. In the example of *C. brevicauda* and *P. aduncum*, based on seed proportions, we infer that 1.5599 g of whole pulp yield 1.0000 g of whole feces. If a conventional gram-to-gram comparison of ingested food and feces is performed, digestive efficiency would be undervalued because the residual nutrients in 1.0000 g of feces originate from >1.0000 g of pulp. Differential changes in the proportions of nutrients, fiber, and seeds further complicate calculations. Therefore: (a) comparisons should not ignore the glucose/protein content of the actual amount of pulp that was ingested to produce the feces; and (b) it is necessary to isolate glucose/protein measurements from changes in the concentration of other pulp/feces components. We achieved both objectives by expressing nutrient content relatively to seed mass, that is, we divided the quantity of glucose/protein in whole pulp by 0.2545 [1.0000 g of whole pulp contain 0.2545 g of seeds], and the quantity of glucose/protein in whole feces by 0.3970 [1.0000 g of whole feces contain 0.3970 g of seeds]. This allowed calculating percent digestive efficiencies^2^ as: *D* = 100 × (*P* − *F*)/*P*, where *P* = quantity (mg) of glucose/protein per gram of seeds in dry pulp, and *F* = quantity (mg) of glucose/protein per gram of seeds in dry feces. We used the same procedure to calculate the digestive efficiency for dry mass.

To reach sound conclusions about assimilation, measures of the quantity nutrients in pulp and feces need to be made comparable. The calculation procedure that we employed for pulp is the implicit standard: *A*_1_ = *nutrient content of whole pulp* = mg of nutrient per g of whole pulp; and *A*_2_ = *nutrient content of pulp without seeds* = mg of nutrient per g of pulp without seeds = *A*_1_/0.7455 [1.0000 g of whole pulp contain 0.7455 g of pulp without seeds]. Intuition inspiring the gram-to-gram comparison would incorrectly assume that *A*_1_ and *A*_2_ are respectively comparable to: *B*_1_ = *nutrient content of whole feces* = mg of nutrient per g of whole feces; and *B*_2_ = *nutrient content of feces without seeds* = mg of nutrient per g of feces without seeds = *B*_1_/0.6030 g [1.0000 g of whole feces contain 0.6030 g of feces without seeds]. Because the nutrient content of a given amount of pulp should be compared with that of the feces resulting from its digestion, *B*_1_ and *B*_2_ must be replaced with: *C*_1_ = *transformed nutrient content of whole feces* = *B*_1_ × 0.6411 [1.0000 g of whole pulp yields 0.6411 g of whole feces]; and *C*_2_ = *transformed nutrient content of feces without seeds* = *B*_2_ × 0.5185 [1.0000 g of pulp without seeds yields 0.5185 g of feces without seeds]. *C*_1_ and *C*_2_ can be defined as the quantity of the nutrient remaining in feces after digestion of 1.0000 gram of pulp. *A*_1_ and *A*_2_ can be used in conjunction with *C*_1_ and *C*_2_ to calculate digestive efficiencies, with identical numerical results (barring rounding errors) to those obtained through the more straightforward procedure described in the preceding paragraph.

Previous authors have estimated percentages of “crude protein” of bat-consumed fruit through multiplication of total nitrogen by a conversion factor of 6.25. We corrected the reported protein estimates by applying a more conservative factor of 4.4^17^. We transformed (Fig. 1; Tables S1, S2, and S3) glucose and protein percentages into mass units (mg/g).

## Supplementary information

Supplementary information

## Data Availability

All data generated or analysed during this study are included in this published article (and its Supplementary information file).
