# Supplementary material for: Small nutrient molecules in fruit fuel efficient digestion and mutualism with plants in frugivorous bats
Source: Sci Rep. 2019 Dec 18;9:19376. doi: 10.1038/s41598-019-55915-z (PMC6920426; doi:10.1038/s41598-019-55915-z)
Supplement: Supplementary file 1 — Supplementary information [file 41598_2019_55915_MOESM1_ESM.pdf]

Supplementary information for:

Small nutrient molecules in fruit fuel efficient digestion and mutualism with plants in frugivorous bats

S. Andrea Moreno[1+], Mariana Gelambi[1], Alejandro Biganzoli[1], and Jesús Molinari[1*]

[1]Departamento de Biología, Facultad de Ciencias, Universidad de Los Andes, Mérida 05101, Venezuela

[+]https://orcid.org/0000-0002-5535-2762

*corresponding author (jmvault@gmail.com), https://orcid.org/0000-0002-9393-5483

Table S1. "Crude protein" (N × 4.4) values based on nitrogen assays (Kjeldahl and Dumas methods) for the pulp of ripe fruit of 110 bat-dispersed plant species. Abbreviations for phyllostomid genera (only Carolliinae and Stenodermatinae are listed) are: *Art* = *Artibeus*, *Car* = *Carollia*, *Der* = *Dermanura*, *Mes* = *Mesophylla*, *Pla* = *Platyrrhinus*, *Pyg* = *Pygoderma*, *Rhi* = *Rhinophylla*, *Stu* = *Sturnira*, *Uro* = *Uroderma*, *Vam* = *Vampyressa*, Uph = unspecified phyllostomids. Abbreviations for pteropodid frugivorous genera are: *Ace* = *Acerodon*, *Cyn* = *Cynopterus*, *Eid* = *Eidolon*, *Epo* = *Epomophorus*, *Not* = *Notopteris*, *Pte* = *Pteropus*, *Rou* = *Rousettus*, Upt = unspecified pteropodids.

Plant family	Plant species	Crude Protein (mg/g)	Bat genera known to consume the fruit	References (N assays)	References (consumption by bats)
Anacardiaceae	*Spondias mombin*	30.27	Art, Car; Eid	1	2, 3, 4, 5, 6, 7
Anacardiaceae	*S. radlkoferi*	82.37	Art, Der	1	3, 8
Annonaceae	*Annona senegalensis*	62.52	Upt	9	10
Annonaceae	*Cananga odorata*	60.72	Not, Pte	11	11, 12, 13
Araceae	*Xanthosoma microrhiza*	102.52	Stu	14	14
Araliaceae	*Schefflera megacarpa*	25.52	Art, Mes, Pla, Uro, Vam	15	15
Arecaceae	*Socratea exorrhiza*	55.88	Art	15	15
Arecaceae	*Syagrus romanzoffiana*	10.49	Art	16	16
Caesalpiniaceae	*Ceratonia siliqua*	45.76	Rou	17, 18	17, 18, 19
Campanulaceae	*Burmeistera cyclostigmata*	69.52	Der, Stu	14	14
Caricaceae	*Carica papaya*	37.40	Eid, Pte	11	7, 11, 12, 20, 21
Clusiaceae	*Vismia angusta*	22.44	Car, Rhi	15	15
Clusiaceae	*V. baccifera*	43.03	Art, Car, Rhi, Stu	22	23, 24, 25
Combretaceae	*Terminalia catappa*	21.38	Art, Pla; Cyn, Eid, Pte	11, 16	4, 5, 7, 11, 12, 16, 20, 26, 27, 28, 29, 30
Cyclanthaceae	*Asplundia peruviana*	48.84	Rhi	15	15
Cyclanthaceae	*Asplundia vagans*	84.04	Stu	14	14
Elaeocarpaceae	*Elaeocarpus ulianus*	42.24	Pte	11	11, 12
Ericaceae	*Arbutus andrachne*	7.74	Rou	18	17, 18
Fabaceae	*Inocarpus fagifer*	80.52	Pte	11	11, 29
Fabaceae	*Prosopis juliflora*	91.20	Cyn	31	31
Gesneriaceae	*Drymonia rubra*	57.64	Der, Stu	14	14
Lecythidaceae	*Barringtonia samoensis*	90.20	Pte	11	11, 12
Lecythidaceae	*Gustavia superba*	107.01	Car	1	32
Loganiaceae	*Fagraea berteroana*	34.32	Pte	11	11, 12
Malvaceae	*Quararibea asterolepis*	38.02	Art, Car	1	3, 6

Marcgraviaceae	*Marcgravia longifolia*	40.48	Rhi	15	15
Melastomaceae	*Conostegia bernoulliana*	29.04	Der	14	14
Meliaceae	*Melia azedarach*	59.05	Epo, Pte, Rou	17, 18	17, 18, 19, 33, 34, 35
Moraceae	*Artocarpus altilis*	42.68	Pte	11	4, 11, 12, 20
Moraceae	*Brosimum alicastrum*	65.47	Art	1	3, 5
Moraceae	*Ficus bengalensis*	24.60	Cyn	31	26, 31
Moraceae	*F. bullenei*	49.98	Uph	1	36
Moraceae	*F. carica*	36.61	Pte, Rou	18	18, 34, 37
Moraceae	*F. costaricana*	48.58	Uph	1	36
Moracaeae	*F. crassiuscula*	25.52	Uph	14	14
Moraceae	*F. exasperata*	77.37	Eid	38, 39	7, 39
Moraceae	*F. fistulosa*	17.60	Cyn, Rou	40	40, 41, 42, 43
Moraceae	*F. glabrata*	67.58	Art	44	6
Moraceae	*F. guaranitica*	47.17	Car	45	45
Moraceae	*F. guianensis*	39.42	Art, Stu	46	47
Moraceae	*F. hispida*	74.80	Cyn, Rou	40	40, 42, 43, 48
Moraceae	*F. insipida*	29.57	Art, Pla	1, 16	2, 3, 16, 49, 50, 51, 52
Moraceae	*F. microcarpa*	31.24	Cyn, Rou	18, 40	18, 40
Moraceae	*F. mucuso*	17.85	Eid	38, 53	7
Moraceae	*F. natalensis*	42.01	Upt	9, 38, 39	54
Moraceae	*F. nymphaeifolia*	4.22	Art, Vam	46	52, 55
Moraceae	*F. obliqua*	51.92	Pte	11	11, 33, 56
Moraceae	*F. obtusifolia*	27.10	Art, Pla	1, 44	2, 3, 51, 57
Moraceae	*F. ovalis*	25.08	Car	57	58
Moraceae	*F. sansibarica*	45.76	Rou	38	19, 54
Moraceae	*F. scabra*	75.68	Pte	11	11, 12
Moraceae	*F.* sp. 1	51.92	Mes, Vam	15	15
Moraceae	*F.* sp. 2	37.40	Art, Uro	15	15
Moraceae	*F. superba*	44.00	Cyn, Pte, Rou	40	33, 40
Moraceae	*F. sur*	57.24	Eid	9, 39	7, 54
Moraceae	*F. sycomorus*	24.64	Epo, Rou	17, 18	17, 18, 19, 54, 59, 60
Moraceae	*F. tinctoria*	69.08	Cyn, Pte	11	11, 43
Moraceae	*F. trichopoda*	47.31	Eid	9	7
Moraceae	*F. trigonata*	39.42	Art	1	3
Moracaeae	*Ficus tuerckheimii*	27.72	Der	14	14

Moraceae	*F. uniauriculata*	81.84	Pte	11	11, 12
Moraceae	*F. variegata*	48.40	Ace, Cyn, Pte, Rou	40	40, 42, 61, 62
Moracaeae	*F. velutina*	73.92	Uph	14	14
Moraceae	*F. yoponensis*	54.78	Art, Der, Stu	1	3, 14
Moraceae	*Maclura tinctoria*	42.68	Art, Car, Stu	57	49, 57, 58, 63,64
Moraceae	*Milicia excelsa*	31.12	Eid	53	7
Moraceae	*Morus nigra*	47.52	Stu; Rou	17, 18	17, 18, 19, 34, 37, 65, 66
Muntingiaceae	*Muntingia calabura*	43.56	Art, Car; Cyn	58	23, 26, 57, 58, 67, 69
Musaceae	*Musa paradisiaca*	49.09	Cyn, Pte	11, 31	4, 11, 31
Myrtaceae	*Eugenia acapulcensis*	44.00	Der	14	14
Myrtaceae	*Psidium guajava*	40.32	Art, Car; Cyn, Eid, Epo, Pte, Rou	11, 31	7, 11, 12, 14, 19, 20, 26, 27, 28, 31, 34, 35, 43, 45, 49, 57, 62, 68, 69, 21
Myrtaceae	*Syzygium cordatum*	41.61	Upt	9	54
Myrtaceae	*S. inophylloides*	19.80	Pte	11	11, 12
Palmaceae	*Phoenix dactylifera*	30.27	Rou	18	18
Piperaceae	*Piper auritum*	81.40	Stu	14	14
Piperaceae	*P. bisasperatum*	54.12	Der, Stu	14	14
Piperaceae	*P. dotanum*	72.16	Der, Stu	14	14
Piperaceae	*P. epigynium*	66.88	Der, Stu	14	14
Piperaceae	*P. gibbosum*	60.28	Stu	14	14
Piperaceae	*P. glabrescens*	92.84	Stu	14	14
Piperaceae	*P. lancaefolium*	34.32	Der, Stu	14	14
Piperaceae	*Pothomorphe umbellata*	51.48	Stu	14	14
Rhamnaceae	*Zizyphus spina-christi*	38.02	Rou	18	18
Rosaceae	*Eriobotrya japonica*	31.68	Epo, Pte, Rou	17, 18	17, 18, 19, 35, 37, 56
Sapotaceae	*Calophyllum inophyllum*	33.44	Cyn, Pte	11	4, 11, 12, 21, 28, 41
Sapotaceae	*Manilkara zapota*	147.80	Art; Cyn, Rou	31	5, 19, 28, 31, 43
Sapotaceae	*Palaquium stehlinii*	40.92	Pte	11	11, 12
Sapotaceae	*Planchonella garberi*	76.56	Pte	11	11, 12
Sapotaceae	*P. samoensis*	86.24	Pte	11	11, 12
Solanaceae	*Acnistus arborescens*	73.48	Uph	14	14
Solanaceae	*Solanum accrescens*	82.28	Uph	14	14
Solanaceae	*S. argenteum*	118.36	Der, Stu	14	14
Solanaceae	*S. atropurpureum*	68.36	Stu	45	70

Solanaceae	*S. brenesii*	73.92	Stu	14	14
Solanaceae	*S. caavurana*	29.92	Art, Car, Stu	16	16, 49
Solanaceae	*S. granuloso-leprosum*	56.08	Art, Car, Pla, Pyg, Stu	16, 45	16, 70, 71, 45
Solanaceae	*S. nudum*	37.40	Der, Stu	14	14
Solanaceae	*S. ochraceo-ferrugineum*	41.36	Uph	14	14
Solanaceae	*S. paniculatum*	59.28	Pla, Stu	45	30, 72
Solanaceae	*S. rugosum*	73.92	Car	22	73
Solanaceae	*S. sisymbrifolium*	35.62	Car	16	16
Solanaceae	*S. trizygum*	113.96	Uph	14	14
Solanaceae	*S. umbellatum*	25.08	Der, Stu	14	14
Solanaceae	*S*. sp. C	73.92	Stu	14	14
Urticaceae	*Cecropia glaziovii*	34.14	Art, Pla, Stu	16	16, 30, 71
Urticaceae	*C. pachystachya*	59.77	Art, Car, Pla, Stu	16, 45	16, 30, 51, 70, 74
Urticaceae	*C. peltata*	41.36	Art, Car, Der, Mes, Stu	58	5, 23, 25, 50, 58, 69, 74
Urticaceae	*C. sciadophylla*	36.52	Art, Car, Pla, Rhi, Stu	15	15, 25, 52, 74
Verbenaceae	*Faradaya amicorum*	50.60	Pte	11	11, 29
Verbenaceae	*Vitex doniana*	12.11	Upt	53	10, 54
	Mean =<br>Min =<br>Max =	**51.47**<br>**4.22**<br>**147.80**			

Phyllostomids only: mean = 53.69; min = 4.22; max = 147.80.

Pteropodids only: mean = 48.89; min = 7.74; max = 147.80.

The fruit of 6 plant species is consumed by both phyllostomids and pteropodids, namely *Manilkara zapota*, *Morus nigra*, *Muntingia calabura*, *Psidium guajava*, *Spondias mombin*, and *Terminalia catappa*.

Table S2. Proteinaceous and total nitrogen content of the fruit pulp of four species of *Piper*. In *P. amalago*, proteinaceous compounds (mg/g) and proteinaceous nitrogen (mg/g) may include protein, peptides, and free amino acids. In the other three species, proteinaceous compounds (mg/g) and proteinaceous nitrogen (mg/g) only include protein. The lower levels of proteinaceous nitrogen (%) in *P. aduncum*, *P. dumosum*, and *P. lanceolatum* is likely attributable to amino acids and peptides being present, but not quantified.

**Species**	**Proteinaceous compounds (mg/g)**	**Proteinaceous nitrogen (mg/g)**	**Total nitrogen (mg/g)**	**Proteinaceous nitrogen (%)**	**Source**
Piper amalago	60.42	10.60	19.30	54.92	58
P. aduncum	2.95	0.52	15.84	3.27	This study, 16, 22, 45
P. dumosum	8.90	1.56	11.50	13.58	15
P. lanceolatum	48.20	8.46	24.00	35.23	15

Proteinaceous nitrogen (mg/g) = proteinaceous compounds (mg/g) / 5.70, following Herbst[58].

Total nitrogen (mg/g) = crude protein (mg/g) / 4.4.

Proteinaceous nitrogen (%) = 100 × proteinaceous nitrogen (mg/g) / total nitrogen (mg/g).

P. amalago. Protein (60.42 mg/g) and crude protein (84.92 mg/g) correspond to A_2.

P. aduncum. Protein (2.95 mg/g) is the average of A_1 (2.52 mg/g) and A_2 (3.38 mg/g), and crude protein (69.71 mg/g) is the average of A (87.93 mg/g), A_1 (66.09 mg/g), and A_2 (55.12 mg/g).

P. dumosum. Protein (8.90 mg/g) and crude protein (50.60 mg/g) correspond to A_2.

P. lanceolatum. Protein (48.20 mg/g) and crude protein (105.60 mg/g) correspond to A_2.

Table S3. Disparity between protein and “crude protein” values for the fruit pulp of 8 pairs of conspecific/congeneric plant species, suggesting protein to be less abundant in pulp than other nitrogen compounds, including free amino acids and peptids. The Bradford method was used for the determination of protein values. The Kjeldahl method was used for the determination of nitrogen values, which were then converted into “crude protein” values based on a hypothetical N × 4.4 relationship.

**Plant family**	**Comparison**	**Protein (mg/g)**	**Crude protein (mg/g)**	**References**
Anacardiaceae	*Spondias lutea* vs. *S. mombin–S. radlkoferi*	0.15	30.27–82.37	1, 75
Apocynaceae	*Hancornia speciosa* vs. *H. speciosa*	0.62	47.06	75, 76
Arecaceae	*Euterpe oleracea* vs. *E. oleracea*	0.20	120.00	75, 77
Cucurbitaceae	*Cucumis melo* vs. *C. melo*	0.24	10.75	75, 78
Malpighiaceae	*Malpighia glabra* vs. *M. glabra*	0.17	141.30	75, 79
Malvaceae	*Theobroma grandiflorum* vs. *T. grandiflorum*	0.54	88.00	75, 80
Myrtaceae	*Eugenia uniflora* vs. *E. stipitata*	0.10	119.00	75, 80
Passifloraceae	*Passiflora* sp. vs. *P. alata*	0.80	13.50	75, 81
	Mean =<br>Min =<br>Max =	**0.35**<br>**0.10**<br>**0.80**	**74.49**<br>**10.75**<br>**141.30**	

Table S4. Mean body masses, intestine lengths, and gastrointestinal transit times for 40 species of bats and small non-flying mammals.

Category/family	Species	Body mass (g)	Intestine length (cm)	Gastrointestinal transit time (min)	References
**Frugivorous bats**					
Phyllostomidae	*Artibeus lituratus*	67.7	47.8	5.2	82, 83, 84
Phyllostomidae	*Carollia brevicauda*	16.5	–	27.0	84, 85
Phyllostomidae	*C. perspicillata*	17.7	20.9	14.3	83, 84, 86, 87, 88
Phyllostomidae	*Sturnira lilium*	19.9	21.8	5.2	83, 84, 87
	Mean =<br>Min =<br>Max =	**30.4**<br>**16.5**<br>**67.7**	**30.2**<br>**20.9**<br>**47.8**	**12.9**<br>**5.2**<br>**27.0**	
**Frugivorous bats**					
Pteropodidae	*Pteropus alecto*	599.3	163	25.3	84, 89
Pteropodidae	*P. poliocephalus*	703.8	169	27.0	84, 89
	Mean =<br>Min =<br>Max =	**651.6**<br>**599.3**<br>**703.8**	**166.0**<br>**163.0**<br>**169.0**	**26.2**<br>**25.3**<br>**27.0**	
**Insectivorous bats**					
Vespertilionidae	*Eptesicus serotinus*	22.4	16.9	88.0	84, 87, 90
Vespertilionidae	*Myotis bechsteini*	8.5	16.8	95.0	84, 90
Vespertilionidae	*M. daubentoni*	7.8	19.0	90.0	84, 87, 90
Vespertilionidae	*M. myotis*	26.8	21.9	55.0	84, 87, 90
Vespertilionidae	*M. nattereri*	7.3	17.6	77.0	84, 87, 90
Vespertilionidae	*Nyctalus leisleri*	13.2	13.7	204.0	84, 90
Vespertilionidae	*N. noctula*	27.2	17.1	70.0	84, 87, 90
Vespertilionidae	*Nyctophilus gouldi*	10.8	–	56.3	84, 91
Vespertilionidae	*Pipistrellus pipistrellus*	5.4	9.9	69.0	84, 90
Vespertilionidae	*Plecotus auritus*	7.2	15.4	92.0	84, 87, 90
Vespertilionidae	*Vespertilio murinus*	21.7	25.5	102.0	84, 87, 90
	Mean =<br>Min =<br>Max =	**14.4**<br>**5.4**<br>**27.2**	**17.4**<br>**9.9**<br>**25.5**	**90.8**<br>**55.0**<br>**204.0**	

**Insectivorous marsupials, shrews**					
Dasyuridae	*Antechinomys laniger*	25.0	–	96.0	92
Dasyuridae	*Planigale gilesi*	9.5	–	30.0	92
Dasyuridae	*P. tenuirostris*	6.0	–	30.0	92
Dasyuridae	*Sminthopsis douglasi*	55.0	–	78.0	92, 93
Soricidae	*Blarina brevicauda*	17.8	46.1	–	94
Soricidae	*Crocidura cyanea*	14.9	11.6	–	95
Soricidae	*C. hirta*	15.5	30.0	–	96
Soricidae	*C. horsfieldi*	6.5	11.0	–	97
Soricidae	*C. suaveolens*	7.4	–	–	98
Soricidae	*Scutisorex somereni*	54.2	41.6	–	99
Soricidae	*Sorex araneus*	8.65	25.4	30.0	100, 101
Soricidae	*S. cinereus*	4.08	21.6	–	94
Soricidae	*S. fumeus*	6.0	23.5	–	94
Soricidae	*S. minutissimus*	2.5	–	25.0	102
Soricidae	*S. palustris*	14.9	33.6	–	94
	Mean =<br>Min =<br>Max =	**16.5**<br>**2.5**<br>**55.0**	**27.2**<br>**11.0**<br>**46.1**	**48.2**<br>**25.0**<br>**96.0**	
**Herbivorous rodents**					
Caviidae	*Cavia porcellus*	437.5	249.7	276.0	103, 104, 105
Chinchillidae	*Chinchilla laniger*	566.5	357.8	400.0	106, 107
Cricetidae	*Lasiopodomys brandtii*	44.8	59.8	45.0	108, 109, 110, 111
Cricetidae	*Microtus townsendii*	57.2	–	93.0	112
Cricetidae	*Phyllotis darwini*	81.0	–	366.0	104
Ctenomyidae	*Ctenomys talarum*	115.9	77.0	262.3	113, 114, 115
Octodontidae	*Octodon degus*	191.8	–	307.5	104, 116, 117, 118, 119, 120
Sciuridae	*Spermophilus columbianus*	543.5	201.5	738.0	94, 112
	Mean =<br>Min =<br>Max =	**254.8**<br>**44.8**<br>**566.5**	**189.2**<br>**59.8**<br>**357.8**	**311.0**<br>**45.0**<br>**738.0**	

Table S5. Digestive efficiencies (%) of 20 bat species. Depending on the study, "efficiency" refers to different measures (absorption, assimilation, digestibility, metabolization, uptake, utilization) that, for the same metabolic currency (sugar, energy, nitrogen, dry matter), tend to be similar in percentage values. Ranges are indicated for digestive efficiencies representing the weighted mean of two or more similar experiments involving the same bat species.

Currency/feeding habits	Experimental diet	Species/data source	*N*	Digestive efficiency	References
**Sugars**					
Frugivorous	Fruit: mashed banana	*Carollia perspicillata*	6	89.2	121
Frugivorous	Sucrose solutions: 10%, 25%	*Epomophorus wahlbergi*	12	97.3 (95.8–98.7)	122
Frugivorous	Fruit: mashed banana	*Sturnira lilium*	2	92.9	121
Insectivorous	Fruit: mashed banana	*Phyllostomus discolor*	6	86.6	121
Nectarivorous	Glucose-fructose-sucrose solution	*Glossophaga longirostris*	1	>99.0	123
			Mean =<br>Min =<br>Max =	**93.0**<br>**86.6**<br>**99.0**	
**Energy**					
Frugivorous	Fruit: *Ficus insipida*	*Artibeus jamaicensis*	6	64.0	124
Frugivorous	Artificial mixture	*A. jamaicensis* *	20	82.4	125
Frugivorous	Artificial mixture	*Carollia perspicillata* *	20	82.4	126
Frugivorous	Fruit: 2 plant species	*Pteropus poliocephalus*	36	84.2 (76.8–91.6)	127
Frugivorous	Fruit: 5 plant species	*Rousettus aegyptiacus*	28	83.0 (54.4–93.9)	17
Frugivorous	Artificial mixture	*R. aegyptiacus* *	20	78.0	125
Insectivorous	Insects: *Tenebrio molitor*	*Lasiurus cinereus*	1	91.0	128
Insectivorous	Insects: *T. molitor*	*Myotis bechsteinii*	35	98.6	129
Insectivorous	Insects: *T. molitor*	*M. daubentonii*	3	89.8	130
Insectivorous	Insects: moths	*M. evotis*	5	78.3	131
Insectivorous	Insects: moths, *T. molitor*	*M. lucifugus*	24	77.2 (74.9–91.2)	131, 132
Insectivorous	Insects: *T. molitor*	*M. nattereri*	27	97.0	129
Insectivorous	Insects: *T. molitor*	*M. thysanodes*	10	90.8	132
Insectivorous	Insects: moths	*M. volans*	4	77.0	131

Insectivorous	Insects: *T. molitor*	*Plecotus auritus*	21	96.7 (89.9–98.8)	129, 130
Nectarivorous	Nectar: 17% honey solution	*Glossophaga commissarisi*	7	99.0	22
Nectarivorous	Fruit: *Piper hispidum*	*G. commissarisi*	7	48.0	22
			Mean =<br>Min =<br>Max =	**83.4**<br>**48.0**<br>**99.0**	
**Protein**					
Hematophagous	Blood: bovine	*Desmodus rotundus*	16	**92.9**	133
**Nitrogen**					
Frugivorous	Artificial mixture	*Artibeus jamaicensis* *	20	49.0	125
Frugivorous	Artificial mixture	*Carollia perspicillata* *	20	67.0	126
Frugivorous	Fruit: 2 plant species	*Pteropus poliocephalus*	55.6	68.4	127
Frugivorous	Artificial mixture	*Rousettus aegyptiacus* *	20	50.0	125
			Mean =<br>Min =<br>Max =	**58.6**<br>**49.0**<br>**68.4**	
**Dry matter** **					
Frugivorous	Artificial mixture	*Artibeus jamaicensis* *	20	81.1	125
Frugivorous	Fruit: 2 plant species	*Carollia perspicillata*	8	57.8 (57.6–58.3)	58
Frugivorous	Artificial mixture	*C. perspicillata* *	20	83.3	126
Frugivorous	Fruit: 2 plant species	*Pteropus poliocephalus*	36	81.4 (69.7–93.0)	127
Frugivorous	Fruit: 5 plant species	*Rousettus aegyptiacus*	27	83.8 (54.2–94.2)	17
Frugivorous	Artificial mixture	*R. aegyptiacus* *	20	77.5	125
Insectivorous	Insects: *Periplaneta Americana*	*Myotis myotis*	9	69.3	134
			Mean =<br>Min =<br>Max =	**76.3**<br>**54.2**<br>**94.2**	

* Canned peaches, protein monkey chow (crude protein), and sucrose.

** A datum (90.8%) for *Desmodus rotundus* (Breidenstein, 1982) is not listed because protein represents ~96% of the dry weight of blood.

Table S6. Data generated during this study for fruit (*Piper aduncum*) and bat feces (*Carollia brevicauda* fed on *P. aduncum* fruit), used to prepare Figure 1, and to conduct the statistical analyses. After digestion, 1.0000 g of whole fruit pulp becomes 0.6411 g of whole bat feces. Likewise, 1.0000 g of fruit pulp without seeds becomes 0.5185 g of feces without seeds. For the rationalization of A_1, A_2, C_1, and C_2, see Methods.

**Type of sample**	**Nutrient**	$\boldsymbol{A_1}$**:** mg/g of the nutrient in 1.0000 g of whole sample	$\boldsymbol{A_2}$**:** mg of the nutrient in 1.0000 g of sample without seeds
Ripe fruit pulp	Glucose	87.2667	117.0631
Ripe fruit pulp	Glucose	93.2562	125.0977
Ripe fruit pulp	Glucose	94.3999	126.6319
Ripe fruit pulp	Glucose	94.9261	127.3378
Ripe fruit pulp	Glucose	95.1300	127.6112
Ripe fruit pulp	Glucose	96.2177	129.0703
Ripe fruit pulp	Glucose	96.3882	129.2990
Ripe fruit pulp	Glucose	96.9081	129.9965
Ripe fruit pulp	Glucose	100.7506	135.1510
Ripe fruit pulp	Glucose	101.4996	136.1557
Ripe fruit pulp	Glucose	102.0496	136.8935
Ripe fruit pulp	Glucose	107.8593	144.6869
Ripe fruit pulp	Glucose	108.4332	145.4568
Ripe fruit pulp	Glucose	110.5380	148.2802
Ripe fruit pulp	Glucose	110.7303	148.5382
Ripe fruit pulp	Glucose	120.2685	161.3331
Ripe fruit pulp	Glucose	122.8803	164.8367
Ripe fruit pulp	Glucose	124.7738	167.3768
Ripe fruit pulp	Glucose	127.0964	170.4924
Ripe fruit pulp	Glucose	127.4965	171.0291
Ripe fruit pulp	Glucose	131.2348	176.0437
Ripe fruit pulp	Glucose	132.2086	177.3500
Ripe fruit pulp	Glucose	134.2482	180.0860
Ripe fruit pulp	Glucose	144.1417	193.3575
Ripe fruit pulp	Glucose	146.5336	196.5662
Ripe fruit pulp	Glucose	168.6260	226.2018
Ripe fruit pulp	Glucose	178.7330	239.7598
Ripe fruit pulp	Glucose	184.9010	248.0338
Ripe fruit pulp	Glucose	244.8036	328.3896
Ripe fruit pulp	Glucose	244.9502	328.5862
Ripe fruit pulp	Protein	1.6673	2.2366
Ripe fruit pulp	Protein	1.8109	2.4292

Ripe fruit pulp	Protein	1.8137	2.4330
Ripe fruit pulp	Protein	1.8211	2.4429
Ripe fruit pulp	Protein	1.8416	2.4705
Ripe fruit pulp	Protein	1.8979	2.5459
Ripe fruit pulp	Protein	2.0158	2.7040
Ripe fruit pulp	Protein	2.0515	2.7520
Ripe fruit pulp	Protein	2.0924	2.8068
Ripe fruit pulp	Protein	2.1342	2.8628
Ripe fruit pulp	Protein	2.1859	2.9323
Ripe fruit pulp	Protein	2.2298	2.9912
Ripe fruit pulp	Protein	2.2531	3.0225
Ripe fruit pulp	Protein	2.3158	3.1065
Ripe fruit pulp	Protein	2.3863	3.2011
Ripe fruit pulp	Protein	2.3925	3.2094
Ripe fruit pulp	Protein	2.4227	3.2499
Ripe fruit pulp	Protein	2.4470	3.2825
Ripe fruit pulp	Protein	2.5309	3.3950
Ripe fruit pulp	Protein	2.5821	3.4637
Ripe fruit pulp	Protein	2.6192	3.5135
Ripe fruit pulp	Protein	2.8507	3.8240
Ripe fruit pulp	Protein	2.8777	3.8603
Ripe fruit pulp	Protein	2.9385	3.9418
Ripe fruit pulp	Protein	3.0816	4.1338
Ripe fruit pulp	Protein	3.1646	4.2451
Ripe fruit pulp	Protein	3.4187	4.5860
Ripe fruit pulp	Protein	3.5081	4.7060
Ripe fruit pulp	Protein	3.8520	5.1673
Ripe fruit pulp	Protein	4.4075	5.9124
**Type of sample**	**Nutrient**	**C_1:** mg of the nutrient in 0.6411 g of whole sample	**C_2:** mg of the nutrient in 0.5185 g of sample without seeds
Bat feces	Glucose	1.8086	2.4261
Bat feces	Glucose	2.5870	3.4702
Bat feces	Glucose	2.9362	3.9387
Bat feces	Glucose	3.7525	5.0337
Bat feces	Glucose	3.8349	5.1444
Bat feces	Glucose	3.9091	5.2439
Bat feces	Glucose	4.7527	6.3754
Bat feces	Glucose	4.9215	6.6019
Bat feces	Glucose	6.2979	8.4482

Bat feces	Glucose	6.4878	8.7030
Bat feces	Glucose	7.6225	10.2251
Bat feces	Glucose	7.6554	10.2693
Bat feces	Glucose	8.3774	11.2378
Bat feces	Glucose	9.1224	12.2372
Bat feces	Glucose	10.9139	14.6403
Bat feces	Glucose	11.1296	14.9297
Bat feces	Glucose	11.2448	15.0843
Bat feces	Glucose	11.3084	15.1696
Bat feces	Glucose	12.3554	16.5740
Bat feces	Glucose	12.6396	16.9553
Bat feces	Glucose	13.3639	17.9269
Bat feces	Glucose	14.8915	19.9761
Bat feces	Glucose	15.0975	20.2524
Bat feces	Glucose	15.8810	21.3034
Bat feces	Glucose	18.0472	24.2092
Bat feces	Glucose	18.4251	24.7161
Bat feces	Glucose	20.3982	27.3630
Bat feces	Protein	0.0167	0.0224
Bat feces	Protein	0.0534	0.0716
Bat feces	Protein	0.0832	0.1116
Bat feces	Protein	0.0896	0.1203
Bat feces	Protein	0.0907	0.1217
Bat feces	Protein	0.1062	0.1424
Bat feces	Protein	0.1112	0.1492
Bat feces	Protein	0.1146	0.1538
Bat feces	Protein	0.1290	0.1731
Bat feces	Protein	0.1375	0.1845
Bat feces	Protein	0.1442	0.1935
Bat feces	Protein	0.1475	0.1979
Bat feces	Protein	0.1624	0.2179
Bat feces	Protein	0.1664	0.2232
Bat feces	Protein	0.1682	0.2257
Bat feces	Protein	0.1721	0.2308
Bat feces	Protein	0.1962	0.2631
Bat feces	Protein	0.1984	0.2661
Bat feces	Protein	0.2084	0.2795
Bat feces	Protein	0.2405	0.3226
Bat feces	Protein	0.2624	0.3520
Bat feces	Protein	0.2642	0.3544
Bat feces	Protein	0.2688	0.3605

Bat feces	Protein	0.3738	0.5015
Bat feces	Protein	0.5482	0.7354
Bat feces	Protein	0.5839	0.7832
Bat feces	Protein	0.6022	0.8079
Bat feces	Protein	0.8671	1.1632
Bat feces	Protein	1.0418	1.3975
Bat feces	Protein	1.9040	2.5541
Bat feces	Protein	2.6973	3.6183

Additional details of biochemical procedures

To extract glucose, we mixed each previously dried and weighted sample with cold 80% ethanol. After using a mortar to carefully mash (without breaking or crushing the seeds, which are very hard anyway) this mixture, we centrifuged the resulting homogenate for 15 min at 5,000 *g* and -4°C, and saved the supernatant. We added cold 80% ethanol to the pellet (consisting a lower layer of seeds, and a upper layer of other solids) so obtained, centrifuged again in the same manner, then separated the new supernatant. We combined the two supernatants obtained in both extractions in a single test tube, which we boiled in a water bath until all the ethanol was eliminated by vaporization.

To quantify glucose, we used a 100 mg/100 mL solution of glucose as the standard. We used a spectrophotometer to record the absorbance of the samples and the standard at 505 nm.

To extract protein, we mixed each previously dried and weighted sample with an extraction buffer (100 mM Hepes, pH 7.0, 0.25 mM NaCl, 4 mM PMSF, 2mM β-mercaptoethanol). After using a mortar to carefully mash (without breaking or crushing the seeds) this mixture, we centrifuged the resulting homogenate for 15 min at 5,000 *g* and -4°C. To precipitate the protein present in the supernatant, we used two volumes of acetonitrile overnight at -4°C. Then, we centrifuged again for 15 min at 5,000 g at room temperature. We discarded the supernatant, and resuspended the pellet in 0.25 M NaCl solution.

To quantify protein, we used solutions of known concentration of bovine serum albumin as standards, and employed a spectrophotometer to record the absorbance of the samples and the standards at 600 nm.

References (Tables S1–S5)

1. Milton, K. Macronutrient patterns of 19 species of Panamanian fruits from Barro Colorado Island. *Neotrop. Primates 15*, 1–7, DOI: https://doi.org/10.1896/044.015.0101 (2008).

2. Estrada, A., Coates-Estrada, R., Vásquez-Yanes, C., & Orozco-Segovia, A. Comparison of frugivory by howling monkeys (*Alouatta palliata*) and bats (*Artibeus jamaicensis*) in the tropical rain forest of Los Tuxtlas, Mexico. *Amer. J. Primatol.* **7**, 3–13, DOI: https://doi.org/10.1002/ajp.1350070103 (1984).

3. Handley, C. O. Jr., Gardner, A. L., & Wilson, D. E. Food Habits. In *Demography and natural history of the common fruit Bat, Artibeus jamaicensis, on Barro Colorado Island, Panama* (Smithsonian Contributions to Zoology no. 511, Washington, 1991).

4. Whittaker, R. J., & Jones, S. H. The role of frugivorous bats and birds in the rebuilding of a tropical forest ecosystem, Krakatau, Indonesia. *J. Biogeogr.* **21**, 245–258, DOI: https://doi.org/10.2307/2845528 (1994).

5. Flores-Martínez, J. J., Ortega, J., & Ibarra-Manríquez, G. El hábito alimentario del murciélago zapotero (*Artibeus jamaicensis*) en Yucatán. *Rev. Mex. Mastozool.* **4**, 22–39 (2000).

6. Andrade, T. Y., Thies, W., Rogeri, P. K., Kalko, E. K. V., & Mello, M. A. R. Hierarchical fruit selection by Neotropical leaf-nosed bats (Chiroptera: Phyllostomidae). *J. Mammal.* **94**, 1094–1101, DOI: https://doi.org/10.1644/12-MAMM-A-244.1 (2013).

7. Abedi-Lartey, M., Dechmann, D. K. N., Wikelski, M., Scharf, A. K., & Fahr, J. Long-distance seed dispersal by straw-coloured fruit bats varies by season and landscape. *Glob. Ecol. Conserv.* **7**, 12–24, DOI: https://doi.org/10.1016/j.gecco.2016.03.005 (2016).

8. López, J. E., & Vaughan, C. Observations on the role of frugivorous bats as seed dispersers in Costa Rican secondary humid forests. *Acta Chiropterol.* **6**, 111–119, DOI: https://doi.org/10.3161/001.006.0109 (2004).

9. Wilson, A.-L., & Downs, C. T. Fruit nutritional composition and non-nutritive traits of indigenous South African tree species. *S. Afr. J. Bot.* **78**, 30–36, DOI: https://doi.org/10.1016/j.sajb.2011.04.008 (2012).

10. Djossa, B. A., Fahr, J., Kalko, E. K.V., & Sinsin, B. A. Fruit selection and effects of seed handling by flying foxes on germination rates of shea trees, a key resource in northern Benin, West Africa. *Ecotropica* **14**, 37–48 (2008).

11. Nelson, S. L., Miller, M. A., Heske, E. J., & Fahey, G. C. Jr. Nutritional consequences of a change in diet from native to agricultural fruits for the Samoan fruit bat. *Ecography* **23**, 393–401, DOI: https://doi.org/10.1111/j.1600-0587.2000.tb00296.x (2000).

12. Banack, S. A. Diet selection and resource use by flying foxes (genus *Pteropus*). *Ecology* **79**, 1949–1967, DOI: https://doi.org/10.2307/176701 (1998).

13. Scanlon, A. T., Petit, S., Tuiwawa, M., & Naikatini, A. High similarity between a bat-serviced plant assemblage and that used by humans. *Biol. Conserv.* **174**, 111–119, DOI: https://doi.org/10.1016/j.biocon.2014.03.023 (2014).

14. Dinerstein, E. Reproductive ecology of fruit bats and the seasonality of fruit production in a Costa Rican cloud forest. *Biotropica* **18**, 307–318, DOI: https://doi.org/10.2307/2388574 (1986).

15. Ripperger, S. P., Heymann, E. W., Tschapka. M., & Kalko, E. K. V. Fruit characteristics associated with fruit preferences in frugivorous bats and saddle-back tamarins in Perú. *Ecotropica* **20**, 53–64 (2014).

16. Batista, C. B., Reis, N. R., & Rezende, M. I. Nutritional content of bat-consumed fruits in a forest fragment in Southern Brazil. *Braz. J. Biol.* **77**, 244–250, DOI: https://doi.org/10.1590/1519-6984.10115 (2017).

17. Korine, C., Arad, Z., & Arieli, A. Nitrogen and energy balance of the fruit bat *Rousettus aegyptiacus* on natural fruit diets. *Physiol. Zool.* **69**, 618–634, DOI: https://doi.org/10.1086/physzool.69.3.30164219 (1996).

18. Korine, C., Izhaki, I., & Arad, Z. Comparison of fruit syndromes between the Egyptian fruit-bat (*Rousettus aegyptiacus*) and birds in East Mediterranean habitats. *Acta Oecol.* **19**, 147–151, DOI: https://doi.org/10.1016/S1146-609X(98)80018-0 (1998).

19. Kulzer, E. Physiological ecology and geographical range in the fruit-eating cave bat genus *Rousettus* Gray 1821 – a review. *Bonn. Zool. Beitr.* **30**, 233–275 (1979).

20. Entwistle, A. C., & Corp, N. The diet of *Pteropus voeltzkowi*, an endangered fruit bat endemic to Pemba Island, Tanzania. *Afr. J. Ecol.* **35**, 351–360, DOI: https://doi.org/10.1111/j.1365-2028.1997.092-8900092.x (1997).

21. Win, S. S., & Mya, K. M. The diet of the Indian flying fox *Pteropus giganteus* (Brünnich. 1782) (Chiroptera: Pteropodidae) in Myanmar - conflicts with local people? *J. Threat. Taxa* **7**, 7568–7572, DOI: https://doi.org/10.11609/JoTT.o4178.7568-72 (2015).

22. Kelm, D. H., Schaer, J., Ortmann, S., Wibbelt, G., Speakman, J. R., & Voigt, C. C. Efficiency of facultative frugivory in the nectar-feeding bat *Glossophaga commissarisi*: the quality of fruits as an alternative food source. *J. Comp. Physiol. B* **178**, 985–996, DOI: https://doi.org/10.1007/s00360-008-0287-3 (2008).

23. Fleming, T. H., & Heithaus, E. R. (Seasonal foraging behavior of the frugivorous bat *Carollia perspicillata*. *J. Mammal.* **67**, 660–671, DOI: https://doi.org/10.2307/1381127 (1986).

24. Molinari, J., & Soriano, P. *Sturnira bidens*. *Mamm. Species* **276**, 1–4, DOI: https://doi.org/10.2307/3504017 (1987).

25. Suárez-Castro, A. F., & Montenegro, O. L. Consumo de plantas pioneras por murciélagos frugívoros en una localidad de la Orinoquía colombiana. *Mastozool. Neotrop.* **22**, 125–139 (2015).

26. Bhat, H. R. Observations on the food and feeding behaviour of *Cynopterus sphinx* Vahl (Chiroptera, Pteropodidae) at Pune, India. *Mammalia* **58**, 363–370, DOI: https://doi.org/10.1515/mamm.1994.58.3.363 (1994).

27. Nyhagen, D. F., Turnbull, S. D., Olesen, J. M., & Jones, C. G. An investigation into the role of the Mauritian flying fox, *Pteropus niger*, in forest regeneration. *Biol. Conserv.* **122**, 491–497, DOI: https://doi.org/10.1016/j.biocon.2004.08.012 (2005).

28. Raghuram, H., Singaravelan, N., Nathan, P. T., Rajan, K. E., & Marimuthu, G. Foraging ecology of pteropodid bats: pollination and seed dispersal. *Bats: Biology, Behavior and Conservation* (Nova Science Publishers, 2011).

29. McConkey, K. R., & Drake, D. R. Low redundancy in seed dispersal within an island frugivore community. *AoB PLANTS*, *7*, plv088; doi:10.1093/aobpla/plv088, DOI: https://doi.org/10.1093/aobpla/plv088 (2015).

30. Silvestre, S. M., da Rocha, P. A., da Cunha, M. A., Santana. J. P., & Ferrari, S. F. Diet and seed dispersal potential of the white-lined bat, *Platyrrhinus lineatus* (E. Geoffroy, 1810), at a site in northeastern Brazil. *Stud. Neotrop. Fauna Environ.* **51**, 37–44, DOI: https://doi.org/10.1080/01650521.2016.1151244 (2016).

31. Ruby, J., Nathan, P. T., Balasingh, J., & Kunz, T. H. Chemical composition of fruits and leaves eaten by short-nosed fruit bat, *Cynopterus sphinx*. *J. Chem. Ecol.* **26**, 2825–2841, DOI: https://doi.org/10.1023/A:1026446011693 (2000).

32. Giannini, N. P., & Kalko, E. K. V. Trophic structure in a large assemblage of phyllostomid bats in Panama. *Oikos* **105**, 209–220, DOI: https://doi.org/10.1111/j.0030-1299.2004.12690.x (2004).

33. Eby, P. An analysis of diet specialization in frugivorous *Pteropus poliocephalus* (Megachiroptera) in Australian subtropical rainforest. *Aust. J. Ecol.* **23**, 443–456, DOI: https://doi.org/10.1111/j.1442-9993.1998.tb00752.x (1998).

34. Mahmood-ul-Hassan, M., Gulraiz, T. L., Rana, S. A., & Javid, A. The diet of Indian flying-foxes (*Pteropus giganteus*) in urban habitats of Pakistan. *Acta Chiropterol.* **12**, 341–347, DOI: https://doi.org/10.3161/150811010X537927 (2010).

35. Jordaan, L. A., Johnson, S. D., & Downs, C. T. Wahlberg's epauletted fruit bat (*Epomophorus wahlbergi*) as a potential dispersal agent for fleshy-fruited invasive alien plants: effects of handling behaviour on seed germination. *Biol. Invasions* **14**, 959–968, DOI: https://doi.org/10.1007/s10530-011-0131-7 (2012).

36. Korine, C., Kalko, E. K. V., & Herre, E. A. Fruit characteristics and factors affecting fruit removal in a Panamanian community of strangler figs. *Oecologia* **123**, 560–568, DOI: https://doi.org/10.1007/PL00008861 (2000).

37. Izhaki, I., Korine, C., & Arad, Z. The effect of bat (*Rousettus aegyptiacus*) dispersal on seed germination in eastern Mediterranean habitats. *Oecologia* **101**, 335–342, DOI: https://doi.org/10.1007/BF00328820 (1995).

38. Conklin, N. L., & Wrangham, R. W. The value of figs to a hind-gut fermenting frugivore: a nutritional analysis. *Biochem. Syst. Ecol.* **22**, 137–151, DOI: https://doi.org/10.1016/0305-1978(94)90004-3 (1994).

39. Kendrick, E. L., Shipley, L. A., Hagerman, A. E., & Kelley, L. M. Fruit and fibre: the nutritional value of figs for a small tropical ruminant, the blue duiker (*Cephalophus monticola*). *Afr. J. Ecol.* **47**, 556–566, DOI: https://doi.org/10.1111/j.1365-2028.2008.00985.x (2009).

40. Corlett, R. T. Characteristics of vertebrate-dispersed fruits in Hong Kong. *J. Trop. Ecol.* **12**, 819–833, DOI: https://doi.org/10.1017/S0266467400010075 (1996).

41. Tan, K. H., Zubaid, A., & Kunz, T. H. Fruit dispersal by the lesser dog-faced fruit bat, *Cynopterus brachyotis* (Muller) (Chiroptera: Pteropodidae). *Malay. Nat. J.* **54**, 57– 62 (2000).

42. Hodgkison, R., Balding, S. T., Zubaid, A., & Kunz, T. H. Fruit bats (Chiroptera: Pteropodidae) as seed dispersers and pollinators in a lowland Malaysian rain forest. *Biotropica* **35**, 491–502, DOI: https://doi.org/10.1111/j.1744-7429.2003.tb00606.x (2003).

43. Mukherjee, A., Wilske, B., & Chen, J. Dietary energy estimate inferred from fruit preferences of *Cynopterus sphinx* (Mammalia: Chiroptera: Pteropodidae) in a flight cage in tropical China. *J. Threat. Taxa* **2**, 908–918, DOI: https://doi.org/10.11609/JoTT.o2326.908-18 (2010).

44. Wendeln, M. C., Runkle, J. R., & Kalko, E. K. V. Nutritional values of 14 fig species and bat feeding preferences in Panama. *Biotropica* **32**, 489–501. DOI: https://doi.org/10.1111/j.1744-7429.2000.tb00495.x (2000).

45. Ricardo, M. C. C. *Germinação de sementes e importância relativa da qualidade, disponibilidade e morfologia de frutos na dieta de Carollia perspicillata (Chiroptera: Phyllostomidae)* (Universidade Estadual Paulista, Botucatu, Ph.D. Thesis, 2013).

46. Simmen, B., & Sabatier, D. Diets of some French Guianan primates: food composition and food choices. *Int. J. Primatol.* **17**, 661–693, DOI: https://doi.org/10.1007/BF02735260 (1996).

47. Novoa, S., Cadenillas, R., & Pacheco, V. Dispersión de semillas por murciélagos frugívoros en bosques del Parque Nacional Cerros De Amotape, Tumbes, Perú. *Mastozool. Neotrop.* **18**, 81–93 (2011).

48. Hodgkison, R., Ayasse, M., Kalko, E. K.V., Häberlein, C., Schulz, S., Mustapha, W. A.W., Zubaid, A., & Kunz, T. H. Chemical ecology of fruit bat foraging behavior in relation to the fruit odors of two species of Paleotropical bat-dispersed figs (*Ficus hispida* and *Ficus scortechinii*). *J. Chem. Ecol.* **33**, 2097–2110, DOI: https://doi.org/10.1007/s10886-007-9367-1 (2007).

49. Mikich, S. B. A dieta dos morcegos frugivoros (Mammalia, Chiroptera, Phyllostomidae) de um pequeno remanescente de Floresta Estacional Semidecidual do sul do Brasil. . *Rev. Bras. Zool.* **19**, 239–249, DOI: https://doi.org/10.1590/S0101-81752002000100023 (2002).

50. Olea-Wagner, A., Lorenzo, C., Naranjo, E., Ortiz, D., & León-Paniagua, L. Diversidad de frutos que consumen tres especies de murcielagos (Chiroptera:Phyllostomidae) en la selva lacandona, Chiapas, Mexico. *Rev. Mex. Biodiv.* **78**, 191–200 (2007).

51. Munin, R. L., Fischer, E., & Gonçalves, F. Food habits and dietary overlap in a phyllostomid bat assemblage in the Pantanal of Brazil. *Acta Chiropterol.* **14**, 195–204, DOI: https://doi.org/10.3161/150811012X654871 (2012).

52. Horsley, T. W. B., Bicknell, J. E., Lim, B. K., & Ammerman, L. K. Seed dispersal by frugivorous bats in Central Guyana and a description of previously unknown plant-animal interactions. *Acta Chiropterol.* **17**, 331–336, DOI: https://doi.org/10.3161/15081109ACC2015.17.2.008 (2015).

53. Rogers, M. E., Maisels, F., Williamson, E. A., Fernandez, M. and & Tutin, C. E. G. Gorilla diet in the Lope Reserve, Gabon: a nutritional analysis. *Oecologia* **84**, 326–339, DOI: https://doi.org/10.1007/BF00329756 (1990).

54. Cumming, G. S., & Bernard, R. T. F. Rainfall, food abundance and timing of parturition in African bats. *Oecologia* **111**, 309–317, DOI: https://doi.org/10.1007/s004420050240 (1997).

55. López, J. E., & Vaughan, C. Food niche overlap among neotropical frugivorous bats in Costa Rica. *Rev. Biol. Trop.* **55**, 301–313 (2007).

56. Markus, N., & Hall, L. Foraging behaviour of the black flying-fox (*Pteropus alecto*) in the urban landscape of Brisbane, Queensland. *Wildl. Res.* **31**, 345–355, DOI: https://doi.org/10.1071/WR01117 (2004).

57. García-Morales, R., Chapa-Vargas, L., Galindo-González, J., & Badano, E. I. Seed dispersal among three different vegetation communities in the Huasteca region, Mexico, analyzed from bat feces. *Acta Chiropterol.* **14**, 357–367, DOI: https://doi.org/10.3161/150811012X661675 (2012).

58. Herbst, L. H. The role of nitrogen from fruit pulp in the nutrition of the frugivorous bat *Carollia perspicillata*. *Biotropica* **18**, 39–44, DOI: https://doi.org/10.2307/2388360 (1986).

59. Fenton, M. B., Brigham, R. M., Mills, A. M., & Rautenbach, I. L. The roosting and foraging areas of *Epomophorus wahlbergi* (Pteropodidae) and *Scotophilus viridis* (Vespertilionidae) in Kruger National Park, South Africa. *J. Mammal.* **66**, 461–468, DOI: https://doi.org/10.2307/1380920 (1985).

60. Bonaccorso, F. J., Winkelmann, J. R., Todd, C. M., & Miles, A. C. Foraging movements of epauletted fruit bats (Pteropodidae) in relation to the distribution of sycamore figs (Moraceae) in Kruger National Park, South Africa. *Acta Chiropterol.* **16**, 41–52, DOI: https://doi.org/10.3161/150811014X683255 (2014).

61. Shilton, L. A., Altringham, J. D., Compton, S. G., & Whittaker, R. J. Old World fruit bats can be long-distance seed dispersers through extended retention of viable seeds in the gut. *Proc. R. Soc. Lond. B* **266**, 219–223, DOI: https://doi.org/10.1098/rspb.1999.0625 (1999).

62. Stier, S. C., & Mildenstein, T. L. Dietary habits of the world's largest bats: the Philippine flying foxes, *Acerodon jubatus* and *Pteropus vampyrus lanensis*. *J. Mammal.* **86**, 719–728, DOI: https://doi.org/10.1644/1545-1542(2005)086[0719:DHOTWL]2.0.CO;2 (2005).

63. Bohlender, E. E., Pérez-Torres, J., Borray-Escalante, N. A., & Stevens, R. D. Dietary variation during reproduction in Seba's short-tailed fruit bat. *J. Mammal.* **99**, 440–449, DOI: https://doi.org/10.1093/jmammal/gyx189 (2018).

64. Bôlla, D. A. S., Carvalho, F., Gazarini, J., Zocche, J. J., & Pedro, W. A. Variação na dieta de *Artibeus lituratus* e *Sturnira lilium* (Chiroptera: Phyllostomidae) em três regiões fitogeográficas no sul do Brasil. *Mastozool. Neotrop.* **25**, 5–16 (2018).

65. Autino, A. G., & Barquez, R. M. Patrones reproductivos y alimenticios de dos especies simpátricas del genero *Sturnira* (Chiroptera, Phyllostomidae). *Mastozool. Neotrop.* **1**, 73–80 (1993).

66. Parry-Jones, K. A., & Augee, M. L. Factors affecting the occupation of a colony site in Sydney, New South Wales by the Grey-headed Flying-fox *Pteropus poliocephalus* (Pteropodidae). *Austral Ecol.* **26**, 47–55, DOI: https://doi.org/10.1046/j.1442-9993.2001.01072.x (2001).

67. Bumrungsri, S., Leelapaibul, W, & Racey, P. A. Resource Partitioning in Sympatric *Cynopterus* bats in Lowland Tropical Rain Forest, Thailand. *Biotropica* **39**, 241–248, DOI: https://doi.org/10.1111/j.1744-7429.2006.00245.x (2007).

68. Stier, S. C. *Dietary habits of two threatened co-roosting flying foxes (Megachiroptera), Subic Bay, Philippines* (University of Montana, Missoula, M.Sc. Thesis, 2003).

69. García-Estrada, C., Damon, A., Sánchez-Hernández, C., Soto-Pinto, L., & Ibarra-Núñez, G. Diets of frugivorous bats in montane rain forest and coffee plantations in southeastern Chiapas, Mexico. *Biotropica* **44**, 394–401, DOI: https://doi.org/10.1111/j.1744-7429.2011.00816.x (2012).

70. Carvalho-Ricardo, M. C., Uieda, W., Fonseca, R. C. B., & Rossi, M. N. Frugivory and the effects of ingestion by bats on the seed germination of three pioneering plants. *Acta Oecol.* **55**, 51e57 (2014).

71. Mello, M. A. R., Kalko, E. K. V., & Silva, W. R. Diet and abundance of the bat *Sturnira lilium* (Chiroptera) in a Brazilian montane Atlantic forest. *J. Mammal.* **89**, 485–492, DOI: https://doi.org/10.1644/06-MAMM-A-411R.1 (2008).

72. Maccarini, V. P., Pastorini, L. H., Bianconi, G. V., & Ortêncio-Filho, H. Digestion time and intactness of seeds ingested by *Sturnira lilium* (E. Geoffroyi, 1810) (Mammalia, Chiroptera). *Stud. Neotrop. Fauna Environ.* **53**, 1–9, DOI: https://doi.org/10.1080/01650521.2017.1357361 (2017).

73. Charles-Dominique, P. Feeding strategy and activity budget of the frugivorous bat *Carollia perspicillata* (Chiroptera: Phyllostomidae) in French Guiana. *J. Trop. Ecol.* **7**, 243–256, DOI: https://doi.org/10.1017/S026646740000540X (1991).

74. Lobova, T. A., Mori, S. A., Blanchard, F., Peckham, H., & Charles-Dominique, P. *Cecropia* as a food resource for bats in French Guiana and the significance of fruit structure in seed dispersal and longevity. *Amer. J. Bot.* **90**, 388–403, DOI: https://doi.org/10.3732/ajb.90.3.388 (2003).

75. Araújo, C. L., Bezerra, I. W. L., Dantas, I. C., Lima, T. V. S., Oliveira, A. S., Miranda, M. R.A., Leite, E. L., & Sales, M. P. Biological activity of proteins from pulps of tropical fruits. *Food Chem.* **85**, 107–110, DOI: https://doi.org/10.1016/j.foodchem.2003.06.010 (2004).

76. Cardoso, L. M., Reis, B. L., Oliveira, D. S., & Pinheiro-Sant'Ana, H. M. Mangaba (*Hancornia speciosa* Gomes) from the Brazilian Cerrado: nutritional value, carotenoids and antioxidant vitamins. *Fruits* **69**, 89–99, DOI: https://doi.org/10.1051/fruits/2013105 (2014).

77. Gordon, A., Cruz, A. P. G., Cabral, L. M. C., de Freitas, S. C., Taxi, C. M. D., Donangelo, C. M., Mattietto, R. A., Friedrich, M., da Matta, V. M., & Marx, F. Chemical characterization and evaluation of antioxidant properties of Açaí fruits (*Euterpe oleracea* Mart.) during ripening. *Food Chem.* **133**, 256–263, DOI: https://doi.org/10.1016/j.foodchem.2011.11.150 (2012).

78. Villanueva, M. J., Tenorio, M. D., Esteban, M. A., & Mendoza, M. C. Compositional changes during ripening of two cultivars of muskmelon fruits. *Food Chem.* **87**, 179–185, DOI: https://doi.org/10.1016/j.foodchem.2003.11.009 (2004).

79. Everitt, J. H. Nutritive value of fruits or seeds of 14 shrub and herb species from south Texas. *Southwest. Nat.* **31**, 101–137, DOI: https://doi.org/10.2307/3670966 (1986).

80. Rogez, H., Buxant, R., Mignolet, E., Souza, J. N. S., Silva, E. M., & Larondelle, Y. Chemical composition of the pulp of three typical Amazonian fruits: araça-boi (*Eugenia stipitata*), bacuri (*Platonia insignis*) and cupuaçu (*Theobroma grandiflorum*). *Eur. Food Res. Technol.* **218**, 380–384, DOI: https://doi.org/10.1007/s00217-003-0853-6 (2004).

81. Souza, V. R., Pereira, P. A.P., Queiroz, F., Borges, S.V., & Carneiro, J. D. S. Determination of bioactive compounds, antioxidant activity and chemical composition of Cerrado Brazilian fruits. *Food Chem.*, **134** 381–386, DOI: https://doi.org/10.1016/j.foodchem.2012.02.191 (2012).

82. Brun, A., Fernández M., G., Price, E. R., Nell, L. A., M. V. Simões, B. M. V., Castellar, A., Gontero-Fourcade, M., Cruz-Neto, A. P., Karasov, W. H., & Caviedes-Vidal, E. Morphological bases for intestinal paracellular absorption in bats and rodents. *J. Morphol*. **280**, 1359–1369, DOI: https://doi.org/10.1002/jmor.21037 (2019).

83. Charles-Dominique, P. Inter-relations between frugivorous vertebrates and pioneer plants: *Cecropia*, birds and bats in French Guyana. *Frugivores and seed dispersal* (Dr. W. Junk Publishers, 1986), DOI: https://doi.org/10.1007/978-94-009-4812-9_12.

84. Moyers-Arévalo, R. L., Amador, L. I., Almeida, F. C., & Giannini, N. P. Data from: Evolution of body mass in bats: insights from a large supermatrix phylogeny. *J. Mamm. Evol.* **2018**, 1–16 (2018).

85. Becker, N. I., Rothenwöhrer, C., & Tschapka, M. Dynamic feeding habits: efficiency of frugivory in a nectarivorous bat. *Can. J. Zool.* **88**, 764–773, DOI: https://doi.org/10.1139/Z10-042 (2010).

86. Baldwin, J. W., & Whitehead, S. R. Fruit secondary compounds mediate the retention time of seeds in the guts of Neotropical fruit bats. *Oecologia* **177**, 453–466, DOI: https://doi.org/10.1007/s00442-014-3096-2 (2015).

87. Jones, K. E., & MacLarnon, A. M. Data from: Affording larger brains: testing hypotheses of mammalian brain evolution on bats. *Am. Nat.*, **164** E20–E31, DOI: https://doi.org/10.1086/421334 (2004).

88. Klite, P. D. Intestinal bacterial flora and transit time of three neotropical bat species. *J. Bacteriol.* **90**, 375–379 (1965).

89. Tedman, R. A., & Hall, L. S. The morphology of the gastrointestinal tract and food transit time in the fruit bats *Pteropus alecto* and *P. poliocephalus* (Megachiroptera). *Aust. J. Zool.* **33**, 625–640, DOI: https://doi.org/10.1071/ZO9850625 (1985).

90. Roswag, A., Becker, N. I., & Encarnação, J. A. Inter- and intraspecific comparisons of retention time in insectivorous bat species (Vespertilionidae). *J. Zool. (Lond.)* **288**, 85–92, DOI: https://doi.org/10.1111/j.1469-7998.2012.00927.x (2012).

91. Grant, J. D. Food-passage time in *Nyctophilus gouldi* (Microchiroptera: Vespertilionidae). *J. Mammal.* **69**, 653–655, DOI: https://doi.org/10.2307/1381367 (1998).

92. Stannard, H. J., & Old, J. M. Rate of passage through the digestive tract of the kultarr (*Antechinomys laniger*). *Aust. J. Zool.*, *59*, 273–276, DOI: https://doi.org/10.1071/ZO11103 (2011).

93. Hume, I. D., Smith, C., & Woolley, P. A. Anatomy and physiology of the gastrointestinal tract of the Julia Creek dunnart, *Sminthopsis douglasi* (Marsupialia: Dasyuridae). *Aust. J. Zool.* **48**, 475–485, DOI: https://doi.org/10.1071/ZO00016 (2000).

94. Schieck, J. O., & Millar, J. S. Alimentary tract measurements as indicators of diets of small mammals. *Mammalia* **49**, 93–104, DOI: https://doi.org/10.1515/mamm.1985.49.1.93 (1985).

95. Boonzaier, J., Van der Merwe, E. L., Bennett, N. C., & Kotze, S. H. Comparative gastrointestinal morphology of three small mammalian insectivores: *Acomys spinosissimus* (Rodentia), *Crocidura cyanea* (Eulipotyphla), and *Amblysomus hottentotus* (Afrosoricida). *J. Morphol*. **274**, 615–626, DOI: https://doi.org/10.1002/jmor.20118 (2013).

96. Korn, H. Intestine lengths of Southern African savanna rodents and insectivores: intra- and interspecific comparisons. *J. Zool. (Lond.)* **228**, 455–460, DOI: https://doi.org/10.1111/j.1469-7998.1992.tb04448.x (1992).

97. Hattori, S., & Yamanouchi, K. Gross anatomy of Watase's shrew, *Crocidura horsfieldi watasei*. *Exp. Anim. (Tokyo)* **33**, 519–524, DOI: https://doi.org/10.1538/expanim1978.33.4_519 (1984).

98. Yuan, L.-l., Peng, X.,Wang, Y., Sun, X.-G., Guan, G.-H., & Yang, M. Mean retention time of digesta through the gut in lesser white-toothed shrew and striped hamster. *Journal of Shenyang Normal University (Natural Science Edition)* **2**, 028 (2008).

99. Churchfield, S., Dieterlen, F., Hutterer, R., & Dudu, A. Feeding ecology of the armored shrew, from the northeastern Democratic Republic of Congo. *J. Zool. (Lond.)* **273**, 40–45, DOI: https://doi.org/10.1111/j.1469-7998.2007.00297.x (2007).

100. Jaroszewska, M., & Wilczyńska, B. Dimensions of surface area of alimentary canal of pregnant and lactating female common shrews. *J. Mammal.* **87**, 589–597, DOI: https://doi.org/10.1644/05-MAMM-A-135R2.1 (2006).

101. Pernetta, J. C. Diets of the shrews *Sorex araneus* L. and *Sorex minutus* L. in Wytham grasslands. *J. Anim. Ecol.* **45**, 899–912, DOI: https://doi.org/10.2307/3588 (1976).

102. Skarén, U. Feeding behaviour, coprophagy and passage of foodstuffs in a captive least shrew. *Acta Theriol.* **23**, 131–140, DOI: https://doi.org/10.4098/AT.arch.78-7 (1978).

103. Gouat, P. Biometrics of the digestive tract of three species of Ctenodactylidae: comparison with other rodents. *Z. Säugetierk.* **58**, 191–193 (1993).

104. Sakaguchi, E., & Ohmura, S. Fibre digestion and digesta retention time in guinea-pigs (*Cavia porcellus*), degus (*Octodon degus*) and leaf-eared mice (*Phyllotis darwini*). *Comp. Biochem. Physiol. A* **103**, 787–791, DOI: https://doi.org/10.1016/0300-9629(92)90182-P (1992).

105. Sakaguchi, E., Itoh, H., Uchida, S., & Horigome, T. Comparison of fibre digestion and digesta retention time between rabbits, guinea-pigs, rats and hamsters. *Br. J. Nutr.* **58**, 149–158, DOI: https://doi.org/10.1079/BJN19870078 (1987).

106. Hagen, K. B., Dittmann, M. T., Ortmann, S., Kreuzer, M., Hatt, J.-M., & Clauss, M. Retention of solute and particle markers in the digestive tract of chinchillas (*Chinchilla laniger*). *J. Anim. Physiol. Anim. Nutr. (Berl.)* **100**, 801-806, DOI: https://doi.org/10.1111/jpn.12441 (2016).

107. Pérez, W., Vázquez, N., & Jerbi, H. Gross anatomy of the intestine and their peritoneal folds in the chinchilla (*Chinchilla lanigera*). *J. Morphol. Sci.* ***28***, 1–4 (2011).

108. Pei, Y.-X., Wang, D.-H., & Hume, I. D. Effects of dietary fibre on digesta passage, nutrient digestibility, and gastrointestinal tract morphology in the granivorous Mongolian Gerbil (*Meriones unguiculatus*). *Physiol. Biochem. Zool.* **74**, 742–749, DOI: https://doi.org/10.1086/322928 (2001).

109. Song, Z. G., & Wang, D. H. Basal metabolic rate and organ size in Brandt's voles (*Lasiopodomys brandtii*): effects of photoperiod, temperature and diet quality. *Physiol. Behav.* **89**, 704–710, DOI: https://doi.org/10.1016/j.physbeh.2006.08.016 (2006).

110. Wang, D.-H., Pei, Y.-X., Yang, J.-C., & Wang, Z.-W. Digestive tract morphology and food habits in six species of rodents. *Folia Zool.* **52**, 51–55 (2003).

111. Zhao, Z.-J., & Wang, D.-H. Effects of diet quality on energy budgets and thermogenesis in Brandt's voles. *Comp. Biochem. Physiol. A* **148**, 168–177, DOI: https://doi.org/10.1016/j.cbpa.2007.04.001 (2007).

112. Hume, I. D., Morgan, K. R., & Kenagy, G. J. Digesta retention and digestive performance in sciurid and microtine rodents: effects of hindgut morphology and body size. *Physiol. Zool.* **66**, 396–411, DOI: https://doi.org/10.1086/physzool.66.3.30163700 (1993).

113. Del Valle, J. C., & López Mañanes, A. A. Digestive flexibility in females of the subterranean rodent *Ctenomys talarum* in their natural habitat. *J. Exp. Zool.* **315**, 141–148, DOI: https://doi.org/10.1002/jez.658 (2011).

114. Martino, N. S., Zenuto, R. R., & Busch, C. Nutritional responses to different diet quality in the subterranean rodent *Ctenomys talarum* (tuco-tucos). *Comp. Biochem. Physiol. A* **147**, 974–982, DOI: https://doi.org/10.1016/j.cbpa.2007.03.003 (2007).

115. Perissinotti, P. P., Antenucci, C. D., Zenuto, R., & Luna, F. Effect of diet quality and soil hardness on metabolic rate in the subterranean rodent *Ctenomys talarum*. *Comp. Biochem. Physiol. A* **154**, 298–307, DOI: https://doi.org/10.1016/j.cbpa.2009.05.013 (2009).

116. Al-kahtani, M. A., Zuleta, C., Caviedes-Vidal, E., & Garland, T. Jr. Kidney mass and relative medullary thickness of rodents in relation to habitat, body size, and phylogeny. *Physiol. Biochem. Zool.* **77**, 346–365, DOI: https://doi.org/10.1086/420941 (2004).

117. Bozinovic, F. Rate of basal metabolism of grazing rodents from different habitats. *J. Mammal.* **73**, 379–384, DOI: https://doi.org/10.2307/1382072 (1992).

118. Edwards, M. S. Nutrition and behavior of degus (*Octodon degus*). *Vet. Clin. N. Am. Exotic Anim. Pract.* **12**, 237–253, DOI: https://doi.org/10.1016/j.cvex.2009.01.003 (2009).

119. Ernest, S. K.M. Life history characteristics of placental nonvolant mammals. *Ecology* **84**, 3402, DOI: https://doi.org/10.1890/02-9002 (2003).

120. Hopkins, S. S. B. Reassessing the mass of exceptionally large rodents using toothrow length and area as proxies for body mass. *J. Mammal.* **89**, 232–243, DOI: https://doi.org/10.1644/06-MAMM-A-306.1 (2008).

121. Laska, M. Food transit times and carbohydrate use in three phyllostomid bat species. *Z. Säugetierk.* **55**, 49–54 (1990).

122. Downs, C. T., Mqokeli, B., & Singh, P. Sugar assimilation and digestive efficiency in Wahlberg's epauletted fruit bat (*Epomophorus wahlbergi*). *Comp. Biochem. Physiol. A* **161**, 344–348, DOI: https://doi.org/10.1016/j.cbpa.2011.12.003 (2012).

123. Winter, Y. In vivo measurement of near maximal rates of nutrient absorption in a mammal. *Journal of Comp. Biochem. Physiol. A* **119**, 853–859, DOI: https://doi.org/10.1016/S1095-6433(98)01026-5 (1998).

124. Morrison, D. W. Efficiency of food utilization by fruit bats. *Oecologia* **45**, 270–273, DOI: https://doi.org/10.1007/BF00346469 (1980).

125. Delorme, M., & Thomas, D. W. Comparative analysis of the digestive efficiency and nitrogen and energy requirements of the phyllostomid fruit-bat (*Artibeus jamaicensis*) and the pteropodid fruit-bat (*Rousettus aegyptiacus*). *J. Comp. Physiol. B* **169**, 123–132, DOI: https://doi.org/10.1007/s003600050202 (1999).

126. Delorme, M., & Thomas, D. W. Nitrogen and energy requirements of the short-tailed fruit bat (*Carollia perspicillata*): fruit bats are not nitrogen constrained. *J. Comp. Physiol. B* **166**, 427–434, DOI: https://doi.org/10.1007/BF02337887 (1996).

127. Steller, D. C. The dietary energy and nitrogen requirements of the grey-headed flying fox, *Pteropus poliocephalus* (Temminck) (Megachiroptera). *Aust. J. Zool.* **34**, 339–349, DOI: https://doi.org/10.1071/ZO9860339 (1986).

128. Brisbin, I. L., Jr. Energy-utilization in a captive hoary bat. *J. Mammal.* **47**, 719–720, DOI: https://doi.org/10.2307/1377909 (1966).

129. Becker, N. I., Encarnação, J. A., Kalko, E. K. V., & Tschapka, M. The effects of reproductive state on digestive efficiency in three sympatric bat species of the same guild. *Comp. Biochem. Physiol. A* **162**, 386–390, DOI: https://doi.org/10.1016/j.cbpa.2012.04.021 (2012).

130. Webb, P. I., Speakman, J. R., & Racey, P. A. Defecation, apparent absorption efficiency, and the importance of water obtained in the food for water balance in captive brown long-eared (*Plecotus auritus*) and Daubenton's *(Myotis daubentoni*) bats. *J. Zool. (Lond.)* **230**, 619–628, DOI: https://doi.org/10.1111/j.1469-7998.1993.tb02710.x (1993).

131. Barclay, R. M., Dolan, M. A., & Dyck, A. The digestive efficiency of insectivorous bats. *Can. J. Zool.* **69**, 1853–1856, DOI: https://doi.org/10.1139/z91-256 (1991).

132. O'Farrell, M. J., Studier, E. H., & Ewing, W. G. Energy utilization and water requirements of captive *Myotis thysanodes* and *Myotis lucifugus* (Chiroptera). *Comp. Biochem. Physiol. A* **39**, 549–552, DOI: https://doi.org/10.1016/0300-9629(71)90318-5 (1971).

133. Breidenstein, C. P. Digestion and assimilation of bovine blood by a vampire bat. *J. Mammal.* **63**, 482–484, DOI: https://doi.org/10.2307/1380446 (1982).

134. Staliński, J. Digestion, defecation and food passage rate in the insectivorous bat *Myotis myotis*. *Acta Theriol.* **39**, 1–11, DOI: https://doi.org/10.4098/AT.arch.94-1 (1994).
